# Innovative methodology for the identification of soluble biomarkers in fresh tissues

**DOI:** 10.18632/oncotarget.24366

**Published:** 2018-01-31

**Authors:** Brunella Costanza, Andrei Turtoi, Akeila Bellahcène, Touko Hirano, Olivier Peulen, Arnaud Blomme, Vincent Hennequière, Eugene Mutijima, Jacques Boniver, Marie-Alice Meuwis, Claire Josse, Benjamin Koopmansch, Karin Segers, Takehiko Yokobori, Karim Fahmy, Marc Thiry, Carla Coimbra, Nancy Garbacki, Alain Colige, Dominique Baiwir, Vincent Bours, Edouard Louis, Olivier Detry, Philippe Delvenne, Masahiko Nishiyama, Vincent Castronovo

**Affiliations:** ^1^ Metastasis Research Laboratory, GIGA Cancer, University of Liège, Liège, Belgium; ^2^ Laboratory for Analytical Instruments, Gunma University Graduate School of Medicine, Gunma, Japan; ^3^ Department of Pathology, University Hospital (CHU), University of Liège, Liège, Belgium; ^4^ Gastroenterology Department, University Hospital (CHU), University of Liège, Liège, Belgium; ^5^ Center for Human Genetic, Molecular Haemato-Oncology Unit, UniLab, University Hospital (CHU), University of Liège, Liège, Belgium; ^6^ Division of Integrated Oncology Research, Research Program for Omics-based Medical Science, Gunma University Initiative for Advanced Research, Gunma, Japan; ^7^ Laboratory of Cell Biology, Faculty of Sciences, University of Liège, Liège, Belgium; ^8^ Department of Abdominal Surgery, University Hospital (CHU), University of Liège, Liège, Belgium; ^9^ Laboratory of Connective Tissues Biology, GIGA-Cancer, University Hospital, University of Liège, Liège, Belgium; ^10^ Mass Spectrometry Laboratory, University of Liège, Liège, Belgium; ^11^ GIGA Proteomics Facility, University of Liège, Liège, Belgium; ^12^ Department of Molecular Pharmacology and Oncology, Gunma University Graduate School of Medicine, Gunma, Japan

**Keywords:** biomarkers, proteomic, miRNAs, tDNA, metabolomic

## Abstract

The identification of diagnostic and prognostic biomarkers from early lesions, measurable in liquid biopsies remains a major challenge, particularly in oncology. Fresh human material of high quality is required for biomarker discovery but is often not available when it is totally required for clinical pathology investigation. Hence, all OMICs studies are done on residual and less clinically relevant biological samples. Here after, we present an innovative, simple, and non-destructive, procedure named EXPEL that uses rapid, pressure-assisted, interstitial fluid extrusion, preserving the specimen for full routine clinical pathology investigation. In the meantime, the technique allows a comprehensive OMICs analysis (proteins, metabolites, miRNAs and DNA). As proof of concept, we have applied EXPEL on freshly collected human colorectal cancer and liver metastases tissues. We demonstrate that the procedure efficiently allows the extraction, within a few minutes, of a wide variety of biomolecules holding diagnostic and prognostic potential while keeping both tissue morphology and antigenicity unaltered. Our method enables, for the first time, both clinicians and scientists to explore identical clinical material regardless of its origin and size, which has a major positive impact on translation to the clinic.

## INTRODUCTION

Biomarkers readily detectable in liquid biopsies are of utmost importance for any healthcare system, in particular for diagnostic and therapeutic applications. This is particularly the case in oncology. The development of high throughput technologies able to perform comprehensive analysis of genes, transcripts, proteins and other significant biological molecules has provided an unprecedented opportunity for the identification of markers of disease processes [1]. Nevertheless, the number of biomarkers approved by the FDA remains modest [2]. Identification of pertinent OMIC biomarkers relies essentially on the availability of patient material ideally collected at different stages of tumour development (e.g. early lesion, before treatment and after recurrence). A major limitation for the discovery of such biomarkers is the inaccessibility of tissue material of interest. Indeed, to this day, it is virtually impossible to access fresh biopsies from pre- or early malignant lesions, as they are required in their entirety for pathological evaluation and diagnosis. The use of liquid biopsies (e.g. serum, urine, saliva) brought the promise to circumvent this problem, opening access to many different entities like circulating DNA, exosomes (containing miRNAs), proteins and metabolites. Indeed, upon their release from the tumour these molecules are diluted up to several billion-fold in blood where they are mixed with species originating from healthy tissues. A “blanket of noise” precludes straightforward analysis and even detection of relevant molecules, covering the biomarkers that would be useful in diagnosing and understanding the disease.

To overcome these difficulties, researchers have focused on tissue interstitial fluid, mainly used for proteomic analysis [3–8]. The available procedures, principally consist in incubating the tissue biopsy in serum-free culture medium for several hours and collecting the liquid and/or in collecting the supernatant after a low speed tissue centrifugation [7]. Both methods, although simple to perform, expose the tissues to long incubations (frequently overnight), which could favour the degradation of diagnostic markers epitopes by endogenous proteolytic enzymes and/or damage the histological architecture. Further to these common and simple methods, more sophisticated approaches based on microdialysis [9] and capillary ultrafiltration exist [10, 11]. They offer the possibility to collect interstitial fluid *in vivo* and *in vitro* but necessitate implantation of the probe in the tissue, most probably leading to distortion of histology. Importantly, none of these methods have ever been evaluated for their compatibility to be integrated in clinical workflow. Consequently, the key problem precluding access to precious tissues that need to be preserved for clinical/histological investigation and diagnosis remains unsolved and represents a major drawback for the identification of clinically relevant biomarkers.

We have developed a simple and original proximal tissue fluid mining method we named EXPEL. It enables efficient extraction of soluble biomarkers while conserving the tissue intact for subsequent pathological analysis. Importantly, the EXPEL method will not only allow the researchers to access human tissues that are very difficult to obtain, but for the first time, scientists and clinicians can share the same material for both experimental research and routine clinical analysis. The emphasis is further laid on the possibility to extract with this method a rich collection of biomolecules such as proteins, metabolites, miRNAs, DNA as well as small vesicles like exosomes. Now, all these moieties are routinely mined for clinical biomarkers aiming at therapeutic and diagnostic applications. An ideal methodology should be rapid to prevent degradation, yet remains comprehensive to extract various molecules in sufficient quantities. We hypothesised that subjecting tissue biopsies to cycles of low-pressure pulses under mild hypertonic conditions would allow a rapid extrusion of interstitial fluid containing the biomarkers of interest, while preserving the morphology and antigenicity of the sample for subsequent pathological investigation. To test the value of the EXPEL method we have applied our procedure to a series of primary colorectal tumours (CRC) and liver metastasis samples (CRC-LM). This proof-of-principle study demonstrates the validity of EXPEL-extruded fluid as unique starting material for the most advanced OMICs methodologies, while showing no disadvantage for routine clinical and pathological investigations.

## RESULTS

### The EXPEL method does not alter tissue morphology and antigenicity

**Figure 1:**
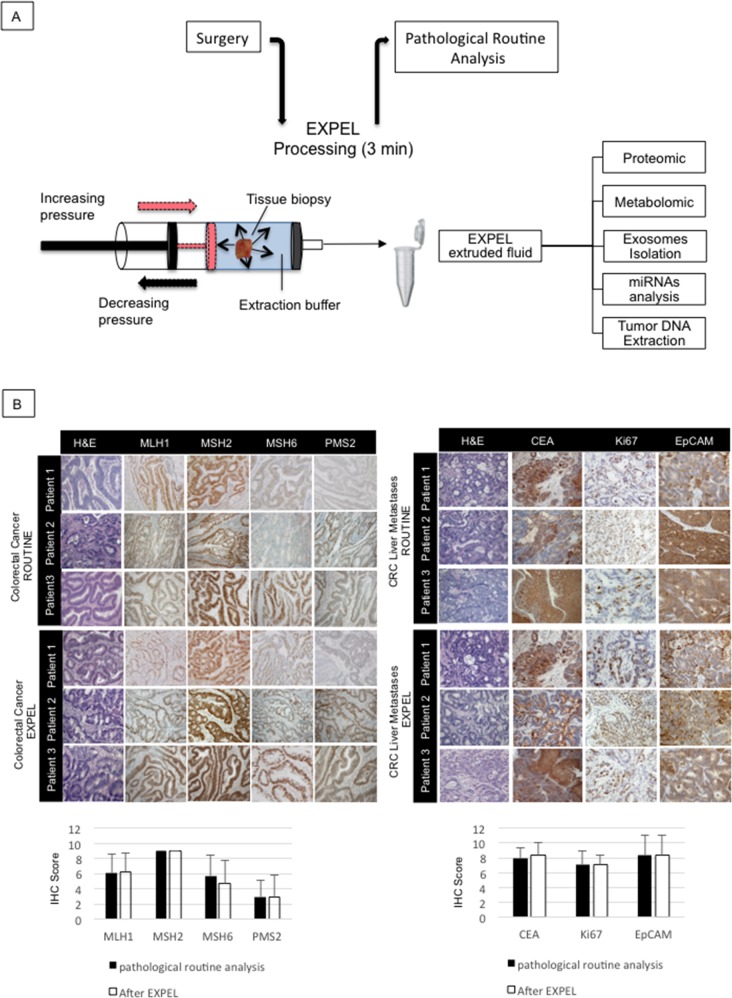
EXPEL method does not alter tissue morphology and antigenicity **(A)** Schematic overview of EXPEL workflow. Diagram of standard tissue processing from the surgery room to pathologists. EXPEL extruded fluid obtained within 3 minutes from a tissue sample, is prepared for the indicated applications or stored at −20°C. **(B)** Colorectal primary tumors (n=10, left panel) and liver metastases (n=10, right panel) were subjected to clinical routine analysis and EXPEL method followed by hematoxylin/eosin (H&E) staining and immunolabelling of the indicated markers (representative pictures are shown for 3 patients). The quantitative evaluation for each marker was assessed as outlined in the Material and Methods section. The error bars indicate standard deviation of means. Images of representative fields were taken at 100× magnification.

To assure that the EXPEL methodology does not alter the morphology and antigenicity of fresh tissue biopsies, we have conducted a comparative analysis of various human and mouse tissues subjected to EXPEL method and standard pathological procedures. For this purpose, we have collected and processed different mouse organs. Each tissue sample was divided into two parts: i) for routine pathological analysis, and ii) for EXPEL processing. A similar workflow was then applied on human breast cancer, colorectal primary tumour and colon cancer liver metastases, together with their adjacent normal counterparts. The routine and EXPEL samples were then subjected to common histology procedures (hematoxylin/eosin) and clinically relevant biomarkers immunodetection. IHC has been conducted using an automated staining platform and three independent pathologists have evaluated stained slides as described under Material and Methods. As displayed in Figure 1 and Supplementary Figure 1, no significant differences were found regarding the tissue structures (H&E) and staining intensities of the selected markers between EXPEL processing and standard pathological sampling method for all analysed human and mouse samples.

### The EXPEL methodology allows the identification of protein biomarkers

**Figure 2:**
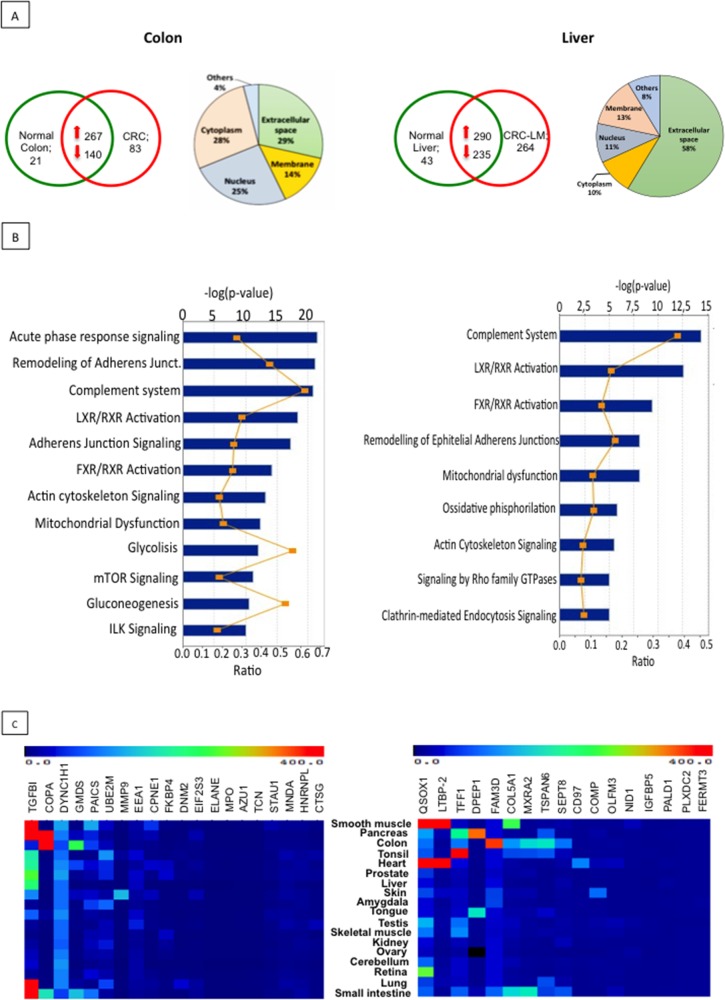
Proteomic analysis of EXPEL extruded fluid identifies potential cancer biomarkers **(A)** Absolute numbers of proteins, identified after proteomic analysis of EXPEL extruded fluids, in at least 4 out of 7 for CRC (upper, left panel) and 3 out of 6 for CRC-LM (upper right, panel) replicates. Pie charts indicating the predicted sub-cellular localization of identified proteins for CRC (on left side) and CRC-LM (on right side) are shown. **(B)** Significantly altered canonical pathways of cancer up-regulated and uniquely expressed proteins analyzed by the IPA software using IPA Core Analysis for colon (on the left) and liver (on the right) EXPEL extruded fluids. The canonical pathways are shown along the y-axis of the bar chart. The x-axis indicates the statistical significance (on the upper part, calculated using the right-tailed Fisher exact test. The P value indicates which biologic annotations are significantly associated with the input molecules relative to all functionally characterized mammalian molecules. “Ratio” (differential yellow line and markers) refers to the number of molecules from the dataset that map to the pathway listed divided by the total number of molecules that map to the canonical pathway from within the IPA knowledgebase. **(C)** Normal tissue gene expression of potential biomarkers candidates discovered from colorectal cancer (left panel) and liver metastases (right panel). mRNA expressions were assessed using BioGPS public database.

EXPEL-extruded fluids were collected from 7 colon cancer and 6 liver metastases patients with their corresponding normal tissue counterparts. They were processed for proteome analysis using UPLC-MS/MS methodology. Due to a difference in complexity, EXPEL fluids derived from colorectal samples were processed using 1D-LC-MS/MS analysis, while those originating from liver metastases were analysed using 2D-LC-MX/MS. Over 2000 and 3000 proteins were identified for colon- and liver- extruded fluids respectively. To obtain an overview of the EXPEL-extruded fluid protein datasets, hierarchical clustering followed by Spearman Rank Correlation was performed using the LFQ (label free quantification, see material and methods) values for both colon and liver samples. Interestingly, as shown in Supplementary Figure 2, the general trend is that EXPEL fluids from the normal samples cluster separately from the tumoural ones. Exceptions to this trend were mainly found in the colon tissues datasets, a result that could be ascribable to the complex cellular composition of these tissues. To narrow down the list of potential biomarkers, we have further considered only proteins consistently identified in at least 4 out of 7 colon- and 3 out of 6 liver- EXPEL-extruded fluids (Figure 2A upper panel, left for CRC and right for CRC-LM). To gain insight in the predicted subcellular localisation of the identified proteins, we have interrogated STRING database (http://string-db.org). Interestingly, we found that the subset of proteins located in the extracellular space was consistently more abundant in comparison with the number of proteins found in other cellular compartments (Figure 2A upper panel pie-charts, left for CRC and right for CRC-LM). This result highlighted the efficiency of the EXPEL method in collecting soluble and accessible proteins. Along with the subcellular localisation we further focused on investigating the pathways that could be altered by the modulated proteins identified in EXPEL fluids from colon and liver patients. To this end, we employed Ingenuity Pathway Analysis tool. The top modulated pathways are shown in Figure 2B (left for CRC and right for CRC-LM) and can be grouped into 3 main classes i) inflammatory response, ii) cytoskeleton reorganisation and iii) metabolism. These 3 classes are well-recognised cancer hallmarks. Following the analysis of differentially expressed proteins, we have examined their expression level in normal healthy tissues. This was done at the mRNA level by interrogating BioGPS public database (Figure 2C, left for CRC and right for CRC-LM). By applying the following criteria, candidate biomarkers could ideally be selected: i) proteins uniquely expressed or consistently overexpressed in the tumour compared to the paired normal tissue, ii) proteins located in the extracellular space and likely to be found in liquid biopsies, particularly plasma, and iii) proteins not or expressed at low levels in normal tissues. Based on these criteria, we have listed in Supplementary Table 3 (for CRC) and Supplementary Table 4 (for CRC-LM), the most promising candidates that deserve future validation tests.

### EXPEL-extruded fluid contains exosomes and miRNAs

**Figure 3:**
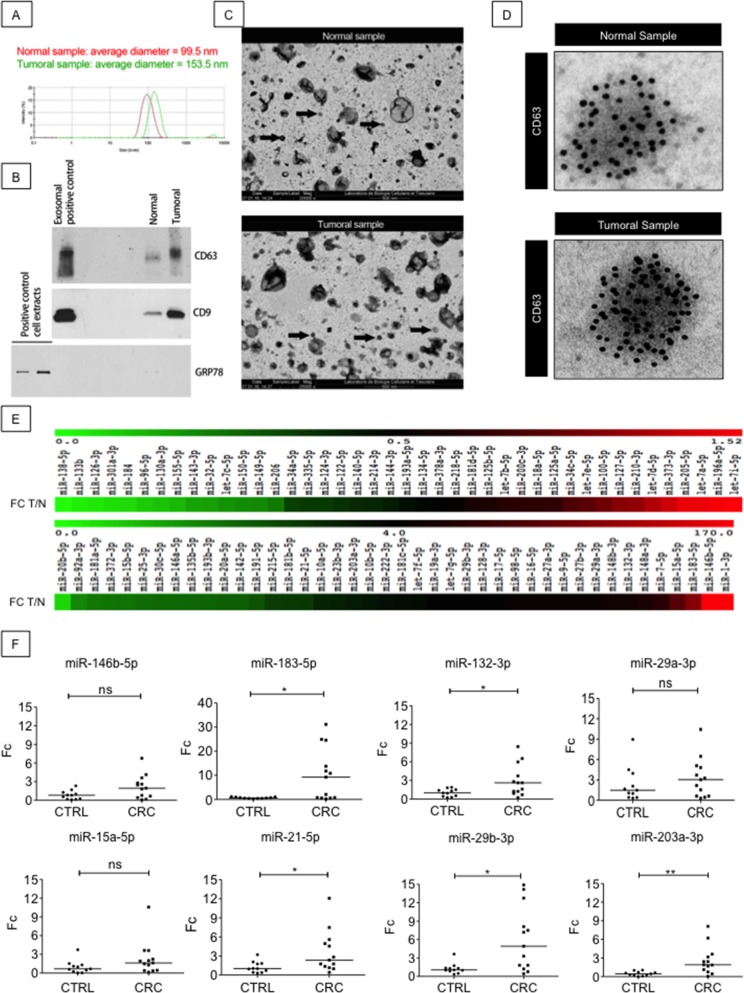
EXPEL extruded fluid contains exosomes and miRNAs readily detectable in patients sera **(A)** Dynamic light scattering of isolated exosomes from normal and tumoral samples revealed that the average diameter of the vesicles is in the expected size range, a representative image of three independent experiment is shown. **(B)** Western blot validation of exosomal preparations. CD9 and CD63 are used as positive control for exosomes isolation whereas GRP78 is employed as control to exclude the presence of contaminating organelles. **(C)** Electron microscopy revealed the presence of exosomes (black arrows) in EXPEL extruded fluid. A representative image of two independent experiments is shown. **(D)** A representative picture of anti-CD63 immunogold labeling evidencing specific staining for exosomal vesicles in normal and tumoral samples. **(E)** miRNome analysis of 84 different miRNAs in EXPEL extruded fluid. The amplified miRNAs are shown in heat maps indicating fold-change differences in tumoral samples versus their normal counterpart (FC T/N). Samples analyzed correspond to a pool of 3 EXPEL extruded fluids from CRC and 3 of their matched normal counterparts. **(F)** Validation of the indicated miRNAs in serum samples of healthy donors (CTRL, n=11) and CRC patients (n=13). Error bars indicate standard deviation of means. Statistical analysis was performed using Whitney U test (^*^ p<0.05, ^**^ p<0.01 and ^***^ p<0.001).

Several experimental evidences point at exosomal proteins and microRNAs (miRNAs) as promising sources of novel biomarkers for clinical diagnosis [12, 13]. Therefore, we asked the question whether EXPEL-extruded fluid could contain exosomes and whether the detection of miRNAs would be possible. We have first attempted to isolate exosomes from EXPEL-extruded fluids of 3 CRC-LM biopsies and their normal counterparts using serial ultracentrifugations. Dynamic light scattering of the isolated vesicles revealed that their average diameter was in the size range expected for exosomes (Figure 3A). To further validate these exosomal preparations, the corresponding protein extracts were resolved by SDS-PAGE. CD9 and CD63 known exosomal markers and GRP78, a protein of the endoplasmic reticulum, were used as controls for exosomes isolation and to exclude presence of contaminating organelles (Figure 3B). We further confirmed the presence of exosomes in EXPEL-derived preparations by transmission electron microscopy. As shown in Figure 3C, the preparations evidenced many vesicles of different sizes, including vesicles with a diameter of less than 100 nm that should correspond to exosomes. Immunogold labeling using anti-CD63 confirmed the presence of exosomes in EXPEL-extruded fluid (Figure 3D).

Encouraged by these findings we next sought to verify whether miRNAs could be detected in EXPEL-extruded fluid. For this purpose, we have isolated RNA from 3 colorectal cancer patients and their corresponding normal tissues. As shown in Figure 3E, EXPEL-extruded fluids allowed the identification and quantification of a discrete panel of cancer-related miRNAs. The differential analysis indicated that many of them were overexpressed in the cancerous lesions by comparison with the normal counterparts and thus could be of particular interest. To further validate our findings and in order to translate our results in a clinical setting, we next verified the expression of 8 selected miRNAs (miR-146b-5p, miR-183-5p, miR-132-3p, miR-29a-3p, miR-15-5p, miR-21-5p, miR-29b-3p, miR-203a-3p) in colorectal cancer patient and healthy donor sera. As shown in Figure 3F, all the miRNAs tested could be amplified by qRT-PCR, therefore rendering the data obtained using EXPEL-extruded fluids readily translatable in a clinical context. Moreover, among the amplified miRNAs, we found that miR-183-5p, miR-132-3p, miR-21-5p, miR-29b-3p, miR-203a-3p were significantly overexpressed in CRC patients compared to healthy donor sera. Further analyses are suggested to assess the use of these miRNAs as signature holding diagnostic potential in the context of CRC.

### EXPEL-extruded fluid contains tumour DNA

**Figure 4:**
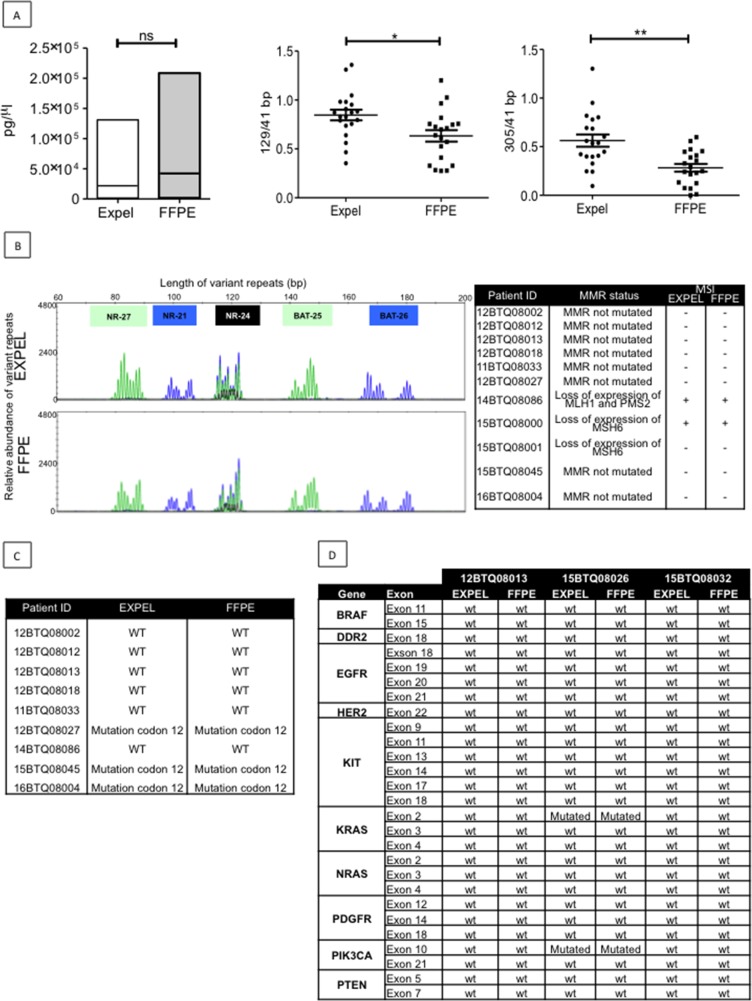
EXPEL extruded fluid contains high quality tumor DNA (tDNA) that is exploitable for genetic profiling **(A)** Left panel, equivalent yield of tDNA was obtained from FFPE sections and matched EXPEL fluids of CRC primary lesions (n=10) and CRC-liver metastatic lesions (n=10). The quality of the DNA was assessed on the same extracts. The ratios of long 129bp (middle panel) and 305bp (right panel) to short amplified fragments (41bp) indicated that EXPEL tDNA presented with significantly higher amounts of long fragments in comparison with the FFPE tDNA. Dot plots show the mean ± SEM. Statistical significance was calculated using Wilcoxon paired test (^*^ p<0.05, ^**^ p<0.01 and ^***^ p<0.001). **(B)** PCR microsatellite instability (MSI) analysis at 5 loci (NR-27, NR-21, NR- 24, BAT-25 and BAT-26) of a representative CRC primary tumor shows similar electropherograms for both FFPE and EXPEL tDNA extracts (left panel). Right panel summarizes MSI analysis results obtained for a subset of 11 tumor samples. The corresponding MMR status, based on routine IHC detection of MMR genes (*MLH1, MSH2, MSH6, PMS2*), is given. **(C)** Concordant KRAS mutational status (codon 12) detected using pyrosequencing on FFPE and EXPEL tDNAs isolated from CRC primary lesions and CRC-liver metastatic lesions. **(D)** Next-generation sequencing (NGS) technique used for the detection of 10 cancer related genetic alterations showed identical results on FFPE and EXPEL tDNAs isolated from CRC and CRC-LM lesions.

Tumour DNA (tDNA) is routinely used as a biomarker for diagnosis and therapy selection in cancer patients [14, 15]. In the clinics, tDNA is extracted from paraffin sections (FFPE) through complex workflow, often yielding small DNA quantities of poor quality. We first examined the possibility that EXPEL-extruded fluids could serve as a source of tDNA and then whether EXPEL tDNA would be comparable in terms of yield and quality to FFPE tDNA. We determined the amounts of FFPE and EXPEL tDNAs from 20 CRC tumour samples including 10 primary lesions and 10 CRC-liver metastases. tDNA was extracted from 20 FFPE slides, according to a routine molecular genetics laboratory protocol (University Hospital of Liège, Belgium) and from a volume of 250 μL of EXPEL-extruded fluid, as described in the Material and Methods section. Under these experimental conditions, we found that the yield of extracted DNA was comparable between FFPE and EXPEL samples (Figure 4A, left panel). Knowing that poor DNA quality mainly has an effect on the amplification of long targets, we next assessed the relative quality of FFPE and EXPEL DNA extracts by normalising 129bp- and 305 bp-fragment concentrations against the concentration obtained for 41 bp-fragments (Figure 4A, middle and right panels, respectively). The ratios of long to short fragments indicated that tDNA extracted from EXPEL fluids presented with significantly better quality (ratios close to 1) in comparison with the tDNA derived from FFPE samples.

Encouraged by these promising results, we next sought to determine if molecular biology tests used in routine CRC diagnostics would match between FFPE and EXPEL tDNA extracts. Nowadays, microsatellite instability (MSI) testing in predicting prognosis and response to chemotherapeutic agents in CRCs is widely used [16]. As shown in Figure 4B (left panel), on a representative case, the MSI-PCR electropherograms at 5 loci (NR-27, NR-21, NR- 24, BAT-25 and BAT-26), were comparable between FFPE and EXPEL tDNAs. All other samples analysed showed concordant MSI positive or negative status (Figure 4B, right panel).

Characterising genomic aberrations in tumours – for diagnosis, therapeutic decisions, and prognostic purposes – using genome-sequencing techniques has become an integral part of the current precision medicine approach [17]. The use of pyrosequencing analysis of KRAS mutational status confirmed the fidelity of EXPEL in comparison to FFPE tDNA extracts, when we searched for codon 12 KRAS mutation (Figure 4C). Next-generation sequencing (NGS) technology has revolutionised the discovery of genetitc markers in cancer research. Soon, NGS will undoubtedly occupy a central position in routine molecular screening of tumours in diagnostic laboratories [18, 19]. Therefore, we next sought to determine whether EXPEL tDNA would be suitable for NGS analysis. As shown in Figure 4D, NGS data obtained for 10 clinically relevant genes using EXPEL tDNA, were fully concordant with those obtained using FFPE tDNA.

### EXPEL-extruded fluids are a rich source of metabolites

**Figure 5:**
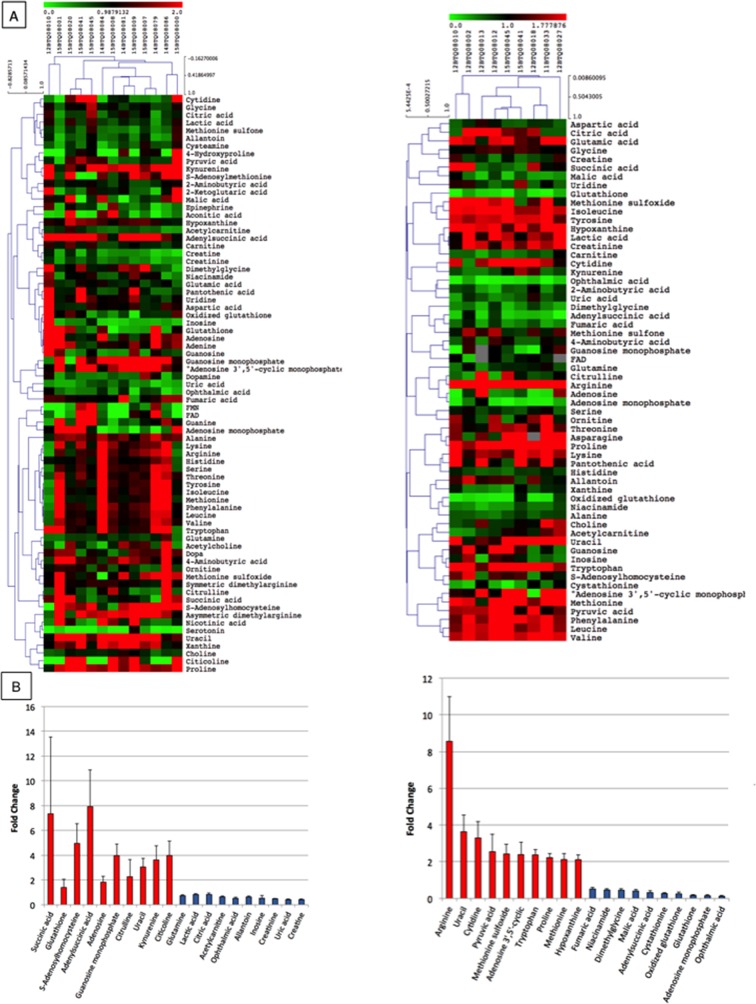
Metabolomic profile of EXPEL extruded fluids from CRC and CRC-LM **(A)** Pearson correlation clustering of general metabolites quantified in the EXPEL extruded fluids from CRC (on the left, n=13) and CRC-LM (on the right, n=9). The individual values are relative quantification ratios of CRC and CRC-LM versus their normal colon and liver adjacent tissues. **(B)** Top 10 up- and down- modulated metabolites are shown for CRC (on the left) and CRC-LM (on the right) EXPEL extruded fluids. Error bars indicate standard error of means.

Considering that cells excrete metabolic byproducts for detoxification and communication purposes [20], and that research is increasingly addressing efforts in cancer metabolome studies as source of biomarkers, we sought to verify whether EXPEL-extruded fluids could be rich in metabolites. Starting from EXPEL-extruded fluid, we have enriched water-soluble metabolites and fatty acid mediators and analysed those using the UPLC-MS (multiple reaction monitoring) approach. As shown in Figure 5A, metabolomic analysis of EXPEL-extruded fluids from both CRC (on the left) and CRC-LM (on the right) allowed a comprehensive detection and quantification of a range of metabolites in pathological samples. In addition, the differential analysis of tumoural samples versus corresponding normal adjacent tissues evidenced a cluster of significantly modulated metabolites, which were detected consistently across all samples (Figure 5B, left for CRC and right for CRC-LM). Among others, elevated levels of succinic acid, S-adenosylhomocysteine and adenylsuccinic acid were readily detected for what concern CRC while arginine was the most abundant metabolite found in the CRC-LM patients. The metabolites consistently upregulated in CRC and CRC-LM analyses were further examined using MetaboAnalyst (www.metaboanalyst.ca). In Supplementary Figure 3 ((A) for CRC and (B) for CRC-LM) are highlighted the metabolic pathways altered in relation to the queried metabolites. Interestingly, we noticed a low level of overlap among the most impacted metabolic pathways between CRC and CRC-LM analyses. This might reflect a potential metabolic reprogramming that occurred during liver metastases dissemination. Along with the analysis of general metabolites, EXPEL-extruded fluids were further used to perform lipidomic profiling. Lipidomic hierarchical clustering analysis followed by Pearson Correlation is shown in Supplementary Figure 4 ((A) left for CRC and panel for CRC-LM) together with the top 20 up- and down- modulated lipid mediators ((B) left for CRC and panel for CRC-LM).

## DISCUSSION

Biomarker discovery is a crucial step for cancer early diagnosis, prognosis and prediction of therapy response. Advances in precision medicine have improved the clinical management of cancer resulting into a better patient clinical outcome while lowering the risks associated to drug toxicity. However, to this day, the identification of new clinically relevant biomarkers for early diagnosis remains disappointing. The traditional approach for biomarker discovery based on a single or a few molecules needs to be revised since it has been shown to be inefficient for complex malignancies like cancer.

In this study, we present EXPEL as a new method for the isolation of molecular species such as proteins, non-coding regulatory RNAs, DNA and metabolites that represent the fundamental material for both researchers and clinicians. The key features of EXPEL include: (a) the simplicity and rapidity of the procedure and its applicability to any tissue regardless of size and origin at no cost; (b) the possibility to share even limited amounts of fresh biopsies between pathologists and researchers; (c) the isolation of diverse biomolecules from a single EXPEL-extruded fluid; (d) the lack of need for optimisation of standard extraction procedures for biomolecules; and (e) the possibility to have long term storage of large collections of frozen EXPEL-extruded fluids allowing their use for prospective follow up and retrospective studies.

This proof-of-concept study was performed on primary human colorectal cancer and colorectal cancer liver metastases. We have demonstrate that the EXPEL methodology leads to the identification of valuable subsets of proteins that could differentiate between cancerous and normal samples. Moreover, the identified modulated proteins were found as strictly linked to cancer disease, thus reducing the probability of obtaining false positive results in following validation steps. Future studies on large series of patients will contribute to validate any of these markers for a clinical use. Apart from proteomic analysis, we have expanded the potential applications of EXPEL-extruded fluids to other OMICs methodologies, including those that are very challenging in terms of quantity and quality of starting biological material, such as miRNome profiling or libraries construction for NGS. For all the applications tested EXPEL-extruded fluids enabled excellent performance and rendered trustable results. In addition, no pre-analytical workflow or purification steps were applied to EXPEL-extruded fluids before any of the analyses were performed. This aspect brings as significant advantage a reduced variability in the pre-analytic workflow favouring more reproducible results among different laboratories. Blood-based test are able to improve patients management for deadly diseases as cancer. It is worth of note that the subset of biomolecules identified in EXPEL-extruded fluids are likely to be found in biological fluids such as serum or plasma. Our research demonstrated it, in the frame of miRNAs study where the identified overexpressed miRNAs were readily detectable in CRC patient sera.

Since the greatest benefits for patients are likely to be reached from the management of early stage disease rather than from treatment of late stage disease, the access to precancerous lesions is essential. However, the clinical workflow imposes that such samples are not shared for research purposes and it directs the researchers towards using residual human material. This practice introduces a sampling bias that is likely to impact on the discovery of clinically qualified biomarkers for cancer diagnosis, prognosis and therapy. Moreover, considering that intratumour heterogeneity has major consequences on treatment design [21], it is critical to perform OMICs analyses and studies on a single tumoural region. To date, and to our knowledge, EXPEL is the first method that enables the simultaneous investigation of wide subset of biomolecules while allowing clinicians and scientists to work in synergy. As such, EXPEL is a timely needed method for systematic sample preparation to acquire high quality catalogues of data following OMICs explorations.

We believe that the EXPEL method opens a new era for biomarkers discovery and will have a major impact on translational research. We trust that this method meets the emerging concept of precision medicine where cancer diagnosis and treatment are tailored to the individual patient characteristics.

## MATERIALS AND METHODS

### Patient material

The ethical committee of the University Hospital of Liege has approved the use of human material in the current study. All fresh and formalin fixed paraffin embedded (FFPE) primary colorectal cancer (CRC) and colorectal liver metastases (CRC-LM) samples were obtained from the institutional Biobank of the University Hospital of Liege, Belgium. According to Belgian law, informed consent was obtained using the opting-out procedure. Following surgical resection, the specimen was immediately transferred to the pathology department where a representative part of the lesion was excised together with the normal counterpart. EXPEL procedure was then applied as described below (Figure 1A). At the end of the procedure the biopsies were fixed in formalin overnight and paraffin-embedded blocks were made for histological evaluations. Serum samples from normal healthy donors and colorectal cancer patients were obtained from the Department of Gastroenterology, University Hospital Liege. Clinical characteristics of individual patients involved in the current study are outlined in Supplementary Table 1 (for tissues) and Supplementary Table 2 (for sera).

### EXPEL-extruded tissue fluids

Fresh surgical biopsies were collected immediately after surgery, cut into 3 mm^3^ pieces and placed in a 10 mL plastic syringe (Cat.: # 302188, BD Plastipak™ Madrid, Spain). One mL of hypertonic extraction buffer (PBS supplemented with NaCl, 4.5 g in 500 mL) was added into the syringe together with the sample. The plunger was then moved to 5 mL mark, allowing an intake of approximately 4 mL of air-bubble between the plunger and the liquid surface. Following this the tissue was subjected to an alternating pressure for 1 min, by moving the plunger from 5 mL to 2.5 mL. Separate measurements with attached manometer showed that the effective pressure ranged between 1.2 bar (compressed state) to 0.8 bar (relaxed state) and 1 bar at (initial condition, plunger at 5 mL mark). The procedure was repeated for a total of three times for each sample. EXPEL-extruded tissue fluids were pooled and stored at −20°C. The whole procedure is completed in 3 min and the EXPEL processed tissue sample can re-enter the classical tissue processing for clinical evaluation.

### Immunohistochemistry (IHC)

Formalin-fixed paraffin-embedded tissue sections were prepared from primary colon tumours, adjacent normal colon as well as from colon cancer liver metastases and their normal liver counterpart. Tissue samples were sliced from paraffin blocks (5 μm sections), deparaffinated 3 times in xylene for 5 min and hydrated in methanol gradient (100%, 95%, 70%, and 50%). Blocking of nonspecific peroxidase activity was conducted for 30 minutes in 3% H_2_O_2_ and 90% methanol. Following this, specimens were first subjected to antigen retrieval in citrate buffer (10 mM, pH 6, 95°C for 45 min) and then blocked in 1.5% normal serum (Vector Laboratories, Burlingame, CA, USA). Tissues were immunostained using following antibodies: CEA (1:8000; Cat.: # GA52661, Dako, Glostrup, Denmark), Ki67 (Cat.: # 790-4286, Ventana Roche, Tucson, AZ, USA), EpCam (Cat.: # 760-4383, Ventana Roche), MLH1 (Cat.: # 790-4535, Ventana Roche), MSH2 (Cat.: # 760-4265, Ventana Roche), MSH6 (Cat.: # 790-4455, Ventana Roche), PMS2 (Cat.:# 760-4531 Ventana Roche). All antibodies were incubated overnight at 4°C. The samples were then washed in PBS and incubated for 30 minutes with biotinylated secondary antibodies (Vector Laboratories, Burlingame, CA, USA). The signal was revealed using avidin-biotin-complex kit (Vectastain ABC kit, Vector Laboratories, Burlingame, CA, USA) and 3,3′-diaminobenzidine tetrachlorhydrate dihydrate (DAB) in 5% H_2_O_2_. The tissues were counter-stained with hematoxylin. IHC scoring was performed by three independent pathologists (EM, JB and PD). Each IHC slide was assessed for the intensity of the staining using the following scale: 0 = no staining, 1 = weak, 2 = moderate and 3 = strong. The tissues were further evaluated for the extent of positivity using the scale: 1 = 0-33%, 2 = 33-66%, 3 = 66-100%. The values obtained by each of the two scales were multiplied to yield a composite value called IHC score.

### Proteomic analysis

Protein quantification of EXPEL-extruded fluids was performed using BCA quantification Kit (Cat.: # 23225, Pierce, Thermo Scientific, Rockford, IL, USA). A volume corresponding to 6 μg of proteins was lyophilised and re-suspended in 50 μL of RapiGest SF surfactant (Cat.: # 186001861, Waters, Waters Corporation, 34 Maple Street, Milford, MA 01757). The samples were incubated for 10 minutes at 90°C. Proteins were further reduced in 1,4-dithiothreitol (10 mM) (Cat.: # D0632-10G, Sigma-Aldrich, St. Louis, MO, USA), for 30 minutes at 60°C and then alkylated using 2-chloroacetamide (22mM) (Cat.: # 30208220, Sigma-Aldrich), for 30 minutes at RT and in darkness. Proteins digestion was conducted overnight at 37°C with trypsin (Cat.: # 29341524, Promega, Madison, WI, USA) using a 1:50 enzyme to protein ratio. Following the digestion, 1μL of PNGase enzyme (Cat.: # P0704S, BioLabs, 240 County Road Ipswich, MA 01938-2723) was added to each preparation and samples were incubated at 37°C for 1h. Peptides were acidified with TFA, at final concentration of 0.5 %, for 1h at 37°C. Samples were further centrifuged at 13000 g for 10 minutes. After collecting the supernatant, peptides were desalted using ZipTip (Cat.: # ZTC18S096, Merck Darmstadt, Germany) according with the manufacturers protocol.

Peptide samples from liver metastases were analysed using 2D-LC-MS/MS analysis, while colorectal samples were analysed using 1D-LC-MS/MS analysis. Before analysis, each sample was spiked with a commercial mixture of protein digest standards originated from non-human biological material: the MassPREP™ Digestion Standards (Waters, Corp., Milford, USA), at 100 and 150 fmol of yeast alcohol dehydrogenase (ADH) for CRC and CRC-LM per sample, respectively. The commercial standard consists of two standard mixtures (MPDS Mix 1 and MPDS Mix 2) containing protein digests of ADH, rabbit glycogen phosphorylase b, bovine serum albumin and yeast enolase at different protein ratios.

### Proteomic analysis of colorectal cancer

one μg of desalted peptides were injected on an Acquity M-Class UPLC (Waters, Milford, MA, USA) connected to a Q Exactive Plus (Thermo Scientific, Bremen, Germany), in nano-electrospray positive ion mode. The samples were loaded on the trap column (Symmetry C18 5μm, 180 μm × 20 mm, Waters) in 100% solvent A (water 0.1% formic acid) during 3 minutes and subsequently separated on the analytical column (HSS T3 C18 1.8 μm, 75 μm × 250 mm, Waters); flow rate 600 nL/min, solvent A (0.1% formic acid in water) and solvent B (0.1% formic acid in acetonitrile), linear gradient 0 min, 98% A; 5 min, 93% A; 135 min, 70% A; 150 min, 60% A, total run time was 180 min. The MS acquisition was conducted in data-dependent mode. The parameters for MS acquisition were: MS range from 400 to 1750 *m/z*, Resolution of 70,000, AGC target of 1e6 or maximum injection time of 50 ms. The parameters for MS2 spectrum acquisition were: isolation window of 2.0 *m/z*, normalised collision energy (NCE) of 25, resolution of 17500, AGC target of 1e5 or maximum injection time of 50 ms, under fill ratio of 1.0%.

### Proteomic analysis of liver metastases

Three μg of peptides were injected onto the 2D-nanoAquity UPLC (Waters, Corp., Milford, USA) coupled online with a Q-Exactive mass spectrometer (Thermo Scientific), operated in nano-electrospray positive ion mode. The 2D-nanoUPLC system consisted of a reversed phase operated at pH 10 and a reversed phase operated at pH 3. The samples were loaded at 2 μL/min (20 mM ammonium formate solution adjusted to pH 10) on the first column (X-Bridge BEH C18 5 μm (300 μm × 50 mm, Waters)) and subsequently eluted in three steps of acetonitrile gradient (13.3%, 19% and 65%). Each eluted fraction was desalted on the trap column (Symmetry C18 5μm (180 μm × 20 mm, Waters)) with previous 10X online dilution to pH 3. The individual fractions were subsequently separated on the analytical column (BEH C18 1.7 μm (75 μm × 250 mm, Waters)) operated at 250 nL/min. The gradient was composed of solvent A (0.1% formic acid in water) and solvent B (0.1% formic acid in acetonitrile) as follows: 0 min, 99% A; 5 min, 93% A; 140 min, 65% A. The total run time of each step was 180 min. The mass spectrometer method is a TopN-MSMS method where N was set to 12, meaning that the spectrometer acquires one Full MS spectrum, selects the 12 most intense peaks in this spectrum (singly charged precursors excluded) and makes a full MS2 spectrum of each of these 12 compounds. The parameters for MS spectrum acquisition were: mass range from 400 to 1750 m/z, resolution at 70000, AGC target of 1e^6^ or maximum injection time of 200 ms. The parameters for MS2 spectrum acquisition were: isolation window of 2.0 m/z, collision energy of 25, resolution of 17500, AGC target of 1e5 or maximum injection time of 50 ms.

### Proteomic data analysis

Raw MS files were analysed by MaxQuant Software (version 1.5.2.8, Max Planck Institute of Biochemistry, Martinsried, Germany). MS/MS spectra were searched against Uniprot human database by the Andromeda search engine. Normalisation of the data was done using LFQ algorithm [22]. Carbamidomethyl (C) was set as a fixed modification, while oxidation of methionine and deammidation of Asn to Asp residues were used as variable modifications for all searches. Detection of minimum two peptides was required for protein identification. The false discovery rate (FDR) was set to 0.01 on the protein and on the peptide levels. The main search tolerance was set at 5 ppm (10 ppm for MS/MS). Additional protein information, such as subcellular localisation, biological functions were determined using gene ontology annotation available on Uniprot (www.uniprot.org), STRING version 10 (www.string-db.org) and ExoCarta (www.exocarta.org). Gene expression level of potential biomarker candidates in healthy human tissues was assessed through BioGPS public mRNA database (www.biogps.org). Pathway analysis of modulated proteins was performed using Ingenuity Pathways Analysis (IPA; Version 8.5; www.ingenuity.com), aberrant functional networks and canonical pathways were recognised. The P value associated with a function or a pathway is a measure of the likelihood that the association between a set of focus genes in the experiment and a given process or pathway is the result of random chance; in general, a P value (calculated using the right-tailed Fisher exact test) <0.05 indicates a statistically significant, non random association.

The mass spectrometry proteomics data have been deposited to the ProteomeXchange Consortium via the PRIDE partner repository with the dataset identifier PXD005693 and PXD005709 for CRC and CRC-LM, respectively.

### Metabolomic analysis of EXPEL-extruded fluid

One mL of methanol (Wako, Tokyo, Japan) was added to 200 μL of EXPEL-extruded fluid. Sampled were then sonicated and split into two separate vials, each containing 0.6 mL of initial solution: i) GM (general metabolites) and ii) LM (lipid mediators). Description of further sample processing was outlined elsewhere [23]. MS data acquisition was performed on UPLC coupled to triple-quadrupole MS (LCMS-8050; Shimadzu, Kyoto, Japan), according to manufacturer instructions for analysing Primary Metabolites version 2.0 (Cat.: # 225-24865A, Shimadzu) and Lipid Mediators version 2.0 (Cat.: # 225-24873A, Shimadzu). Peak areas of individual metabolites were normalised against the spiked internal standards and then uploaded in the Multi Experiment Viewer software version 4.8 [24] and subjected to Pearson correlation clustering.

### Exosomes isolation from EXPEL-extruded fluid

Exosomes were isolated from EXPEL-extruded fluids using differential ultracentrifugation as previously described [25]. Briefly, fresh EXPEL-extruded fluids of colorectal cancer liver metastasis and normal adjacent liver were filtered (0.22 μm filter) to remove debris. The filtered fluids were then ultracentrifuged for 2 hours at 100000 g (SW-28 rotor, Optima LE80 ultracentrifuge, Beckman Coulter, Brea, CA, USA). The exosome pellets were washed once with PBS, centrifuged again for 2 hours at 100000 g and finally suspended in 80 μl PBS. Sample quality was assessed from 5 μl by dynamic light scattering with Zetasizer Nano ZS (Malvern Instruments, Ltd., Worcestershire, UK). Remaining sample was used for transmission electron microscopy analysis or western-blot protein detection. Proteins were isolated from exosome suspensions with 1% SDS lysing buffer supplemented with protease and phosphatase inhibitors. Protein concentration was assessed by bicinchoninic acid assay (Cat.: # 23225; Pierce, Rockford, IL, USA). Labeling of exosomes for quality control purposes was performed as previously described [26]. Briefly, exosomes adsorbed on formvar-carbon coated grids were successively washed, fixed in 2% formaldehyde and incubated for 2 hours at RT with primary anti-CD63 antibody (Cat.: # ab124045, Abcam) diluted 1/20 in PBS-BSA (0.2%) supplemented with normal goat serum (1/50) and 1 hour at RT with gold-conjugated secondary anti-rabbit antibody (Aurion, Wageningen, The Netherlands) diluted 1/40 in PBS-BSA (0.2%, pH 8.2). Samples were postfixed for 10 minutes in 2.5% glutaraldehyde and contrasted using uranyl acetate and lead citrate. Pictures were made with a Jeol JEM-1400 transmission electron microscope at 80 kV (Jeol, Peabody, MA, USA).

**Table:** 

miRNA ID	Accession Number	Sequence
HSA-miR-15a-5p	MIMAT0000068	UAGCAGCACAUAAUGGUUUGUG
HSA-miR-132-3p	MIMAT0000426	UAACAGUCUACAGCCAUGGUCG
HSA-miR-29a-3p	MIMAT0000086	UAGCACCAUCUGAAAUCGGUUA
HSA-miR-146b-5p	MIMAT0002809	UGAGAACUGAAUUCCAUAGGCU
HSA-miR-183-5p	MIMAT0000261	UAUGGCACUGGUAGAAUUCACU
HSA-miR-21-5p	MIMAT0000076	UAGCUUAUCAGACUGAUGUUGA
HSA-miR-29b-3p	MIMAT0000100	UAGCACCAUUUGAAAUCAGUGUU
HSA-miR-203a-3p	MIMAT0000264	GUGAAAUGUUUAGGACCACUAG
HSA-miR-16-5p	MIMAT0000069	UAGCAGCACGUAAAUAUUGGCG
Cel-miR-39-3p	MIMAT0000010	UCACCGGGUGUAAAUCAGCUUG

### miRNA isolation, miRNome profile and qPCR from EXPEL-extruded fluid

RNA was isolated from EXPEL-extruded fluids and human sera using miRNeasy Serum/Plasmakit (Cat.: # 217184, Qiagen, Hilden, Germany), according to the manufacturer protocol. Before extraction, C. elegans miR-39 (Cat.: # 219610, Qiagen) was spiked in each individual sample (1.6 × 10^8^ copies/μL) as internal control. For miRNome profiling (discovery), the extracted RNA from 3 colorectal patients were pooled to have one sample for the tumoural and one sample for normal tissues. For targeted qPCR (validation) all patient sera were processed individually. In both cases, 5 μL of RNA were used as template for a RT reaction using miScriptII RT kit (Cat.: # 218161, Qiagen) according with manufacturer protocol. The cDNA was further diluted 1:5 in RNase-free water and 5 μL of diluted samples were used as template for the pre-amplification reaction using miScript PreAMP PCR Kit (Cat.: # 331452, Qiagen). For the pre-amplification reaction for EXPEL and sera samples, miScript PreAMP Pathway Primer Mix (Cat.: # 331241, Qiagen) and a mix of the primers listed below were used according with manufacturer's protocol. Preamplified samples were diluted 1:20 in RNase-free water (except from the normal colon sample that was used undiluted). EXPEL-extruded fluid samples were used for miRNome analysis using miScript miRNA PCR Array (Human Cancer PathwayFinder Cat.: # 331221), while the sera samples were checked for the miRNAs listed in the table below. All qPCRs were done using miScript SYBR Green PCR Kit (Cat.: # 218073, Qiagen) according to manufacturer instructions.

### DNA isolation from EXPEL-extruded fluid

DNA was isolated from EXPEL-extruded fluid and paraffin embedded sections from colorectal cancer tumours and liver metastasis samples. Briefly, DNA was extracted from 250 μl of EXPEL-extruded fluids using the QIAamp Circulating Nucleic Acid Kit (Cat.: # 55114, Qiagen) following the manufacturer's protocol. FFPE tissues were cut in 4 μm sections and mounted on glass slides. In order to maximise tumoural content, cancerous tissues areas were defined by a pathologist and manually macro-dissected from 20 tissue slides. DNA extraction was then obtained using an AllPrep DNA/RNA FFPE extraction kit (Cat.: # 80234, Qiagen) according to manufacturer's protocol.

### Microsatellite instability

DNA was extracted from a subset of FFPE samples and their corresponding EXPEL-extruded fluids as described above. MSI-PCR testing was performed using multiplex PCR assay comprising 5 quasimonomorphic mononucleotide repeats (BAT-26, BAT-25, NR-21, NR-22 and NR-24) as previously described [27]. Samples demonstrating instability for at least 3 of the 5 mononucleotide markers evaluated were considered as MSI positive. Known MSI positive and negative DNA samples were tested in parallel as controls.

### PCR for DNA quantification and quality assessment

Absolute DNA quantification and quality assessment based on qPCR experiments were performed using the LightCycler480 qPCR machine (Roche Diagnostics) and KAPA hgDNA Quantification and QC Kit (Cat.: # KK4963, Sopachem, Belgium) according to the manufacturer's protocol. Briefly, a pre-diluted set of DNA standards and primer targeting different portions of a highly conserved single-copy human locus are amplified in an optimised SYBR Green I-based qPCR. Absolute quantification is achieved with the primer pair defining the shortest fragment (41bp), whereas the additional primers amplifying 129bp and 305bp fragments are used to derive information about the amount of amplifiable template in the DNA sample. Since poor DNA quality has a greater impact on the amplification of longer targets, the relative quality of a DNA sample can be inferred by normalising the concentration obtained using the 129 bp or 305 bp assay against the concentration obtained from the 41 bp assay.

### Pyrosequencing analysis

DNA was extracted from a subset of FFPE samples and their corresponding EXPEL-extruded fluids as described above. PCR and pyrosequencing were performed for KRAS codon 12 region as previously described [28].

### Next-generation sequencing

DNA was extracted from FFPE samples and their corresponding EXPEL-extruded fluids as described above. The indicated regions of interest were amplified using a laboratory developed multiplex PCR using Qiagen multiplex PCR plus kit (Qiagen). Molecular barcoding was performed with ‘MID for Illumina Miseq’ (Multiplicom, Belgium) according to the manufacturer's protocol. PCR products of each patient were the pooled and purified using Agencourt AMPure XP beads (Beckman Coulter, USA). Each patient library was then quantified using ‘Quanti-it Picogreen dsDNA Assay Kit’ (Fisher Scientific, USA) and the Fluostar Optima System (BMG Labtech, Germany). Equimolar amounts of each patient PCR products were then pooled to obtain the final library. The library was then quantified and denatured according to the standard Illumina's MiSeq protocol. Sequencing was performed with a MiSeq v2 cartridge with 500 cycles (Illumina, USA). Alignment of the fastq files and variant calling were made using SeqNext Version 4.1.1 build 511 (JSI medical systems, Germany).

### Statistical analysis

Data were analysed using the GraphPad Prism 4 software program. For miRNA studies statistical significance of the expression was assessed using Mann–Whitney *U*-test. tDNA results were analysed according with Wilcoxon paired test. A value of P≤ 0.05 was considered to be significant.

## SUPPLEMENTARY MATERIALS FIGURES AND TABLES










