# Supplementary material for: Innovative methodology for the identification of soluble biomarkers in fresh tissues
Source: Oncotarget. 2018 Jan 31;9(12):10665–80. doi: 10.18632/oncotarget.24366 (PMC5828218; doi:10.18632/oncotarget.24366)
Supplement: Supplementary file 1 [file oncotarget-09-10665-s001.pdf]

Innovative methodology for the identification of soluble biomarkers in fresh tissues

SUPPLEMENTARY MATERIALS

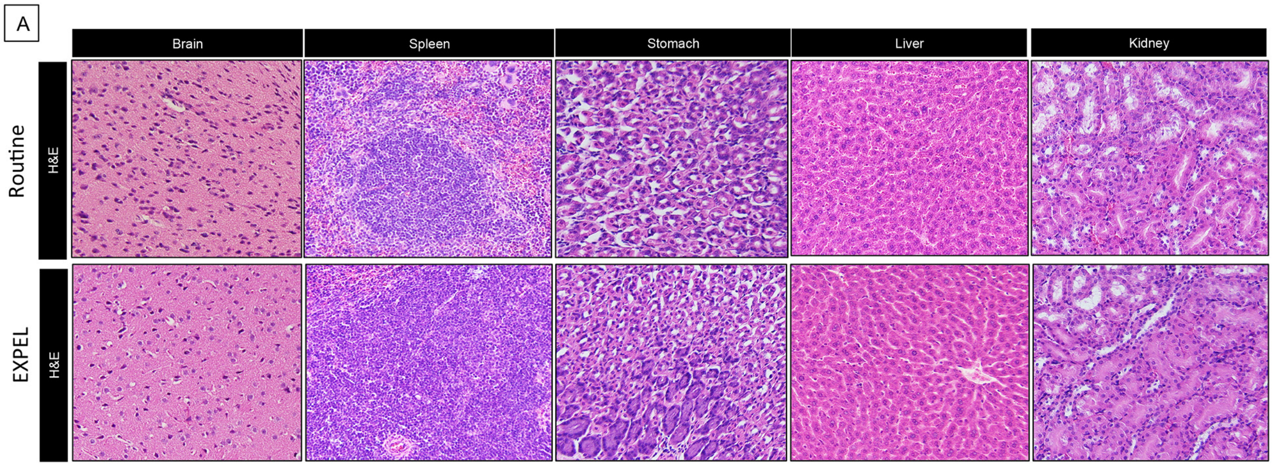

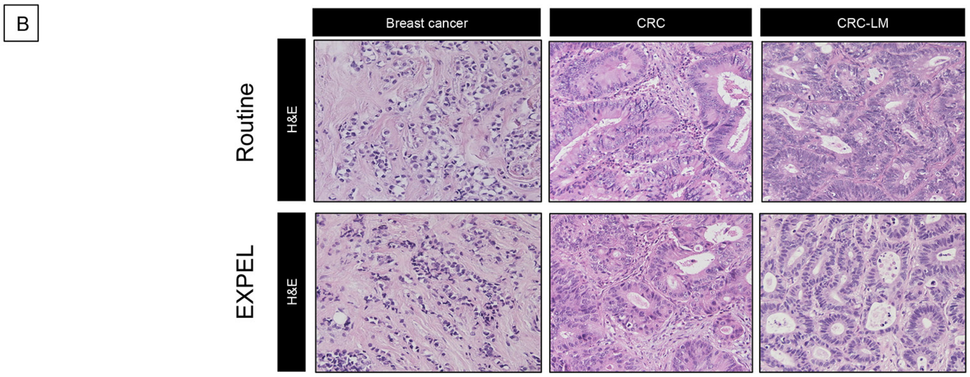

Supplementary Figure 1: EXPEL method does not alter tissue morphology and it can be applied to every tissue. **(A)** Hematoxylin/eosin staining on FFPE sections of the indicated mouse organs subjected either to routine or EXPEL processing. **(B)** Representative images of hematoxylin/eosin staining performed on FFPE sections of breast cancer (n=3), colorectal cancer (CRC; n=3), colorectal cancer liver metastasis (CRC-LM; n=3) subjected either to routine or EXPEL processing. Three pathologists evaluated tissue morphology independently. Images of representative fields were taken at 100× magnifications.

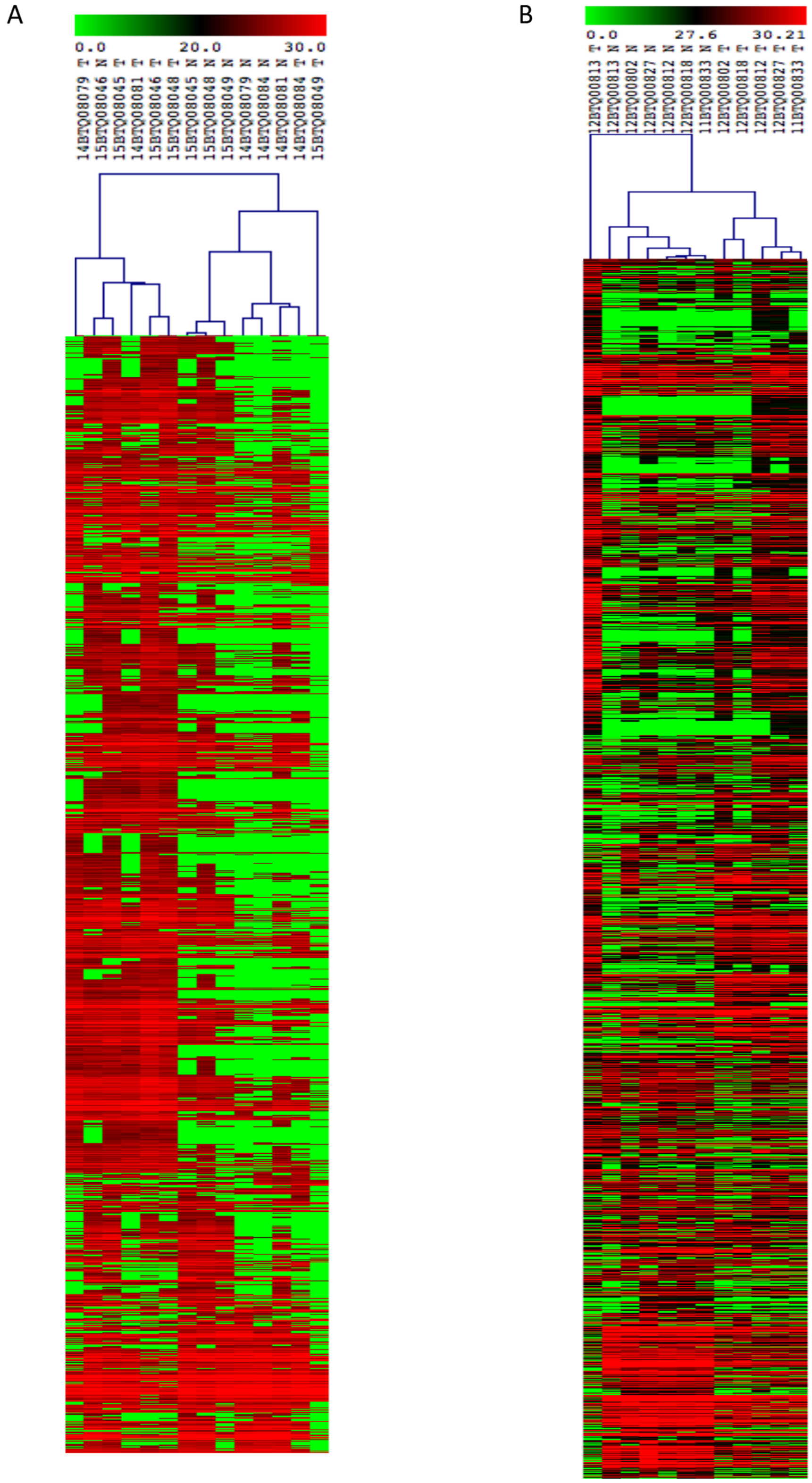

Supplementary Figure 2: Proteomic profile of EXPEL extruded fluids. Hierarchical clustering followed by Spearman Rank Correlation is shown for (**A**) colon (n=7, pairs match) and (**B**) liver (n=6, pairs match) EXPEL extruded fluids.

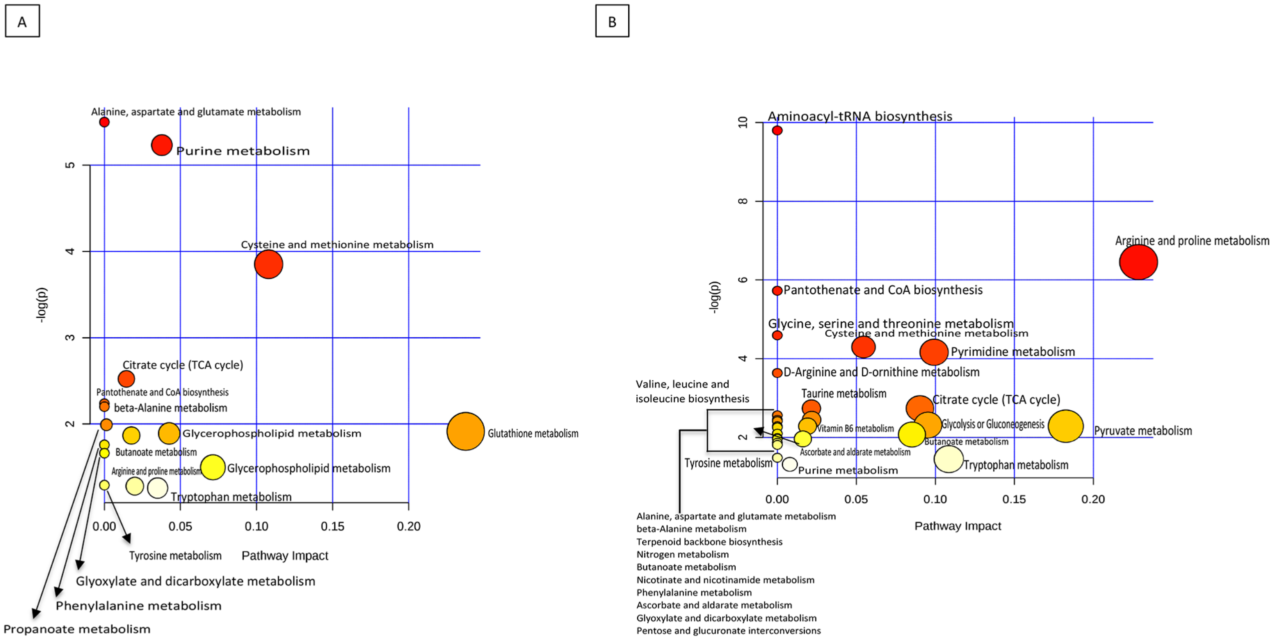

Supplementary Figure 3: Metabolic alteration of EXPEL extruded fluids. Topology-based pathway analysis showing metabolic networks potentially regulated in relation with the most abundant metabolites detected for (**A**) CRC and (**B**) CRC-LM EXPEL extruded fluids. The most impacted metabolic pathways are specified by the volume and the color of the spheres (yellow, least relevant; red, most relevant) according to their statistical relevance P and impact value.

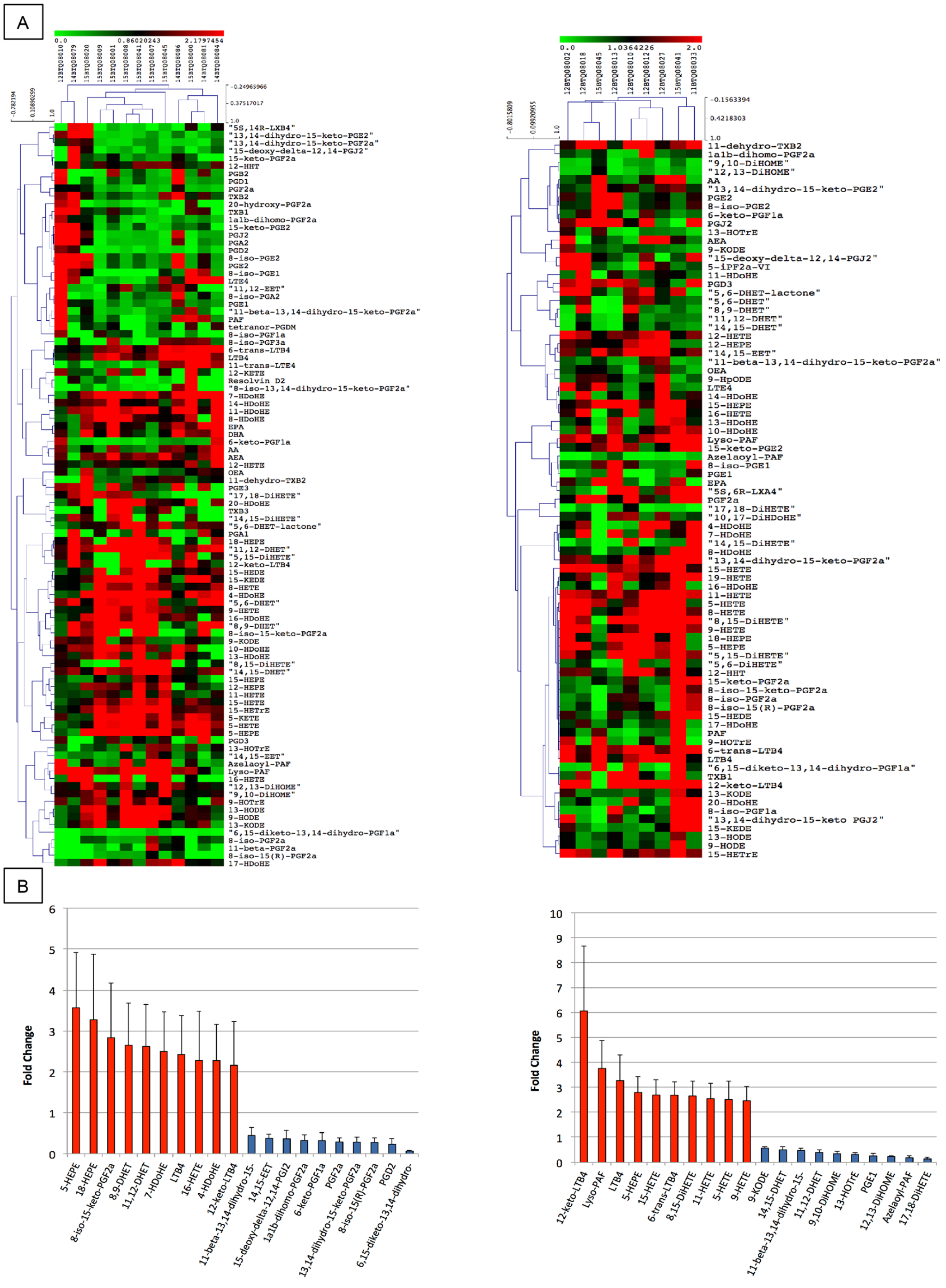

Supplementary Figure 4: Lipidomic analysis of EXPEL extruded fluids from CRC and CRC-LM patients. (A) Pearson correlation clustering of lipid mediators quantified in EXPEL fluids from CRC (on the left, n=13) and CRC-LM (on the right, n=9). The individual values are relative quantification ratios of CRC and CRC-LM versus their normal colon and liver adjacent tissues. **(B)** Top 10 up- and down- modulated lipid mediators are shown for CRC (on the left) and CRC-LM (on the right). Error bars indicate standard error of means.

Supplementary Table 1: Patients clinical characteristics

See Supplementary File 1

Supplementary Table 2: Patients clinical informations

See Supplementary File 2

Supplementary Table 3: Candidate protein biomarkers for CRC

See Supplementary File 3

Supplementary Table 4: Candidate protein biomarkers for CRC-LM

See Supplementary File 4
