# Supplementary material for: Innovative methodology for the identification of soluble biomarkers in fresh tissues
Source: Oncotarget. 2018 Jan 31;9(12):10665–80. doi: 10.18632/oncotarget.24366 (PMC5828218; doi:10.18632/oncotarget.24366)
Supplement: Supplementary file 2 [file oncotarget-09-10665-s002.pdf]

Table S1. Patients clinical characteristics

Patients ID	Tissue type	Surgery date	TNM	Tumor type	Outcome	Kras Status	MMR	Proteomic	Metabolomic	Exosomes isolation	miRNome	tDNA analysis
12BTQ08010	CRC; CRC-LM	May-12	pT3N2bM1a	ADK moderately to poorly differentiated	Alive	wt	Not mutated	✗	✓✓	✗	✗	✓
14BTQ08079	CRC	Nov-14	pT4bN0Mx	ADK moderately differentiated	Alive	Not tested	Not mutated	✓	✓	✗	✗	✗
14BTQ08081	CRC	Nov-14	pT3N2aMx	ADK moderately differentiated	Alive	Not tested	Not mutated	✓	✓	✗	✗	✗
14BTQ08084	CRC	Dec-14	pT4aN2aMx	ADK moderately to poorly differentiated	Alive	wt	Not mutated	✗	✓	✗	✗	✓
14BTQ08086	CRC	Dec-14	pT3 N1b Mx	ADK colloid moderately differentiated	Alive	Not tested	Loss of expression of MLH1 and PMS2	✗	✓	✗	✗	✓
15BTQ08000	CRC	Jan-15	pT3N0Mx	ADK moderately to poorly differentiated	Alive	Not tested	Loss of expression of MSH-6	✓	✓	✗	✗	✓
15BTQ08001	CRC	Jan-15	pT3N1Mx	ADK moderately differentiated	Alive	Not tested	Loss of expression of MSH-6	✗	✓	✗	✗	✓
15BTQ08007	CRC	Jan-15	pT3N0Mx	ADK moderately differentiated	Alive	Not tested	Not mutated	✗	✓	✗	✗	✗
15BTQ08008	CRC	Jan-15	pT3N2bMx	ADK infiltrating moderately differentiated	Alive	Not tested	Not mutated	✗	✓	✗	✗	✗
15BTQ08009	CRC	Jan-15	pT3N2aMx	ADK moderately differentiated	Alive	Not tested	Not mutated	✗	✓	✗	✗	✗
15BTQ08026	CRC	Mar-15	pT4b N2b M1b	ADK colloid moderately differentiated	Dead	Mutation codon 12	Not tested	✗	✓	✗	✗	✓
15BTQ08041	CRC; CRC-LM	Jun-15	pT3N0M1a	ADK moderately differentiated	Alive	wt	Not mutated	✗	✓✓	✗	✗	✓
15BTQ08045	CRC; CRC-LM	Jun-15	pT3N1bM1a	ADK moderately differentiated	Alive	Mutation codon 12	Not mutated	✓	✓✓	✓	✓	✓
15BTQ08046	CRC; CRC-LM	Jun-2015; Dec-2015	pT4bN2nMx	ADK colloid poorly to moderately differentiated	Alive	Not tested	Not mutated	✓	✗	✓	✓	✓
15BTQ08048	CRC	Jun-15	pT3N0Mx.	ADK infiltrating moderately differentiated	Alive	Not tested	Not mutated	✓	✗	✗	✗	✗
15BTQ08049	CRC	Jun-15	pT4aN0Mx	ADK mucinous	Alive	Not tested	Not mutated	✓	✗	✗	✓	✗
15BTQ08053	CRC	Jul-15	T4bN1M1b.	ADK moderately differentiated	Alive	Mutation codon 13	Loss of expression of MLH1 and PMS2	✗	✗	✗	✗	✓
16BTQ08000	CRC	Jan-16	pT4aN2aM1a	ADK poorly differentiated	Alive	wt	Loss of expression of MLH1 and PMS3	✗	✗	✗	✗	✓
16BTQ08001	CRC	Jan-16	pT3N2cM1a	ADK moderately differentiated	Alive	Not tested	Not mutated	✗	✗	✗	✗	✓
12BTQ08002	CRC-LM	Jan-12	–	ADK of colon	Alive	wt	Not mutated	✓	✓	✗	✗	✓
12BTQ08018	CRC-LM	Sep-12	–	ADK of Ileum-colon	Alive	wt	Not mutated	✓	✓	✗	✗	✓
11BTQ08033	CRC-LM	Jun-11	–	ADK rectum	Alive	wt	Not mutated	✓	✓	✗	✗	✓
12BTQ08012	CRC-LM	Jun-12	–	ADK of the rectosigmoid junction	Alive	wt	Not mutated	✓	✓	✗	✗	✓
12BTQ08027	CRC-LM	Nov-12	–	ADK recto-sigmoide	Alive	Mutation codon 12	Not mutated	✓	✓	✗	✗	✓
12BTQ08013	CRC-LM	Jun-12	–	Sigmoidectomy for ADK (Dec 2007); ADK ileo-colon (20/06/2008)	Dead	wt	Not mutated	✓	✓	✗	✗	✓
15BTQ08032	CRC-LM	Apr-15	–	ADK of colon	Alive	wt	Not tested	✗	✓	✗	✗	✓
16BTQ08004	CRC-LM	Jan-16	–	ADK of colon	Alive	Mutation codon 12	Not mutated	✗	✗	✓	✗	✓

CRC: colorectal cancer
CRC-LM: colorectal cancer liver metastasis
ADK: adenocarcinoma
