# Supplementary material for: Innovative methodology for the identification of soluble biomarkers in fresh tissues
Source: Oncotarget. 2018 Jan 31;9(12):10665–80. doi: 10.18632/oncotarget.24366 (PMC5828218; doi:10.18632/oncotarget.24366)
Supplement: Supplementary file 3 [file oncotarget-09-10665-s003.pdf]

Table S2. Patients clinical informations

Patient ID	Gender	Sampling date	Age	Pathology	Localization	Status of the patient	TNM	MS status
1	F	6/6/12	90	ADK	Rectum	Naive	pT3N0M1	NR
2	M	21/06/2012 and 25/06/12	76	ADK	Rectum	Naive	pT3N1M0	NR
3	F	8/8/12	73	ADK	Right colon	Naive	pT4aN0M1a	MSI
4	M	10/23/12	80	ADK	Rectum	Under treatment	pT3NxM1	MSS
5	F	10/31/12	60	ADK	Right colon	Naive	pT3N0Mx	MSS
6	F	11/6/12	76	ADK	Sigmoide	Under treatment	pT3N2aMx	MSS
7	F	11/8/12	81	ADK	Right colon	Naive	pT3N0Mx	MSI
8	M	23/7/2013	69	ADK	Rectum	Naive	pT3NxMx	NR
9	F	26/6/2013	84	ADK	Right colon	Naive	pT4bN0Mx	NR
10	F	26/6/2013	85	ADK	Sigmoide	Naive	pT4aN1aM1b	NR
11	F	5/9/13	75	ADK	Sigmoide	Naive	pT4bN1bM1a	NR
12	F	5/17/13	67	ADK	Right colon	Under treatment	pT4aN0Mx	MSS
13	F	5/17/13	80	ADK	Rectum	Under treatment	pT4bN0Mx	MSS
14	F	26/6/2013	71	ADK	Sigmoide	Under treatment	pT4aN0Mx	MSI
15	F	12/7/13	76	ADK	Rectum	Under treatment	pT4N3M0	NR
16	F	27/8/2013	33	ADK	Left colon	Under treatment	pT4bN1bM1a	MSS
17	M	7/23/13	52	ADK	Sigmoide	Under treatment	pT4aN2aM+	MSS
18	F	11/6/12	64	Healthy CTRL				
19	M	1/23/13	58	Healthy CTRL				
20	F	1/23/13	63	Healthy CTRL				
21	M	1/15/13	78	Healthy CTRL				
22	F	10/26/12	60	Healthy CTRL				
23	F	11/14/12	63	Healthy CTRL				
24	M	10/15/12	43	Healthy CTRL				
25	F	12/14/12	66	Healthy CTRL				
26	F	9/17/13	70	Healthy CTRL				
27	F	2/4/15	68	Healthy CTRL				
28	F	11/26/12	65	Healthy CTRL				
29	M	1/16/13	52	Healthy CTRL				
30	F	5/5/14	68	Healthy CTRL				
31	F	8/24/46	67	Healthy CTRL				
32	F	10/3/14	65	Healthy CTRL				

MSS: microsatellite stable
MSI: microsatellite instable
