# Supplementary material for: Innovative methodology for the identification of soluble biomarkers in fresh tissues
Source: Oncotarget. 2018 Jan 31;9(12):10665–80. doi: 10.18632/oncotarget.24366 (PMC5828218; doi:10.18632/oncotarget.24366)
Supplement: Supplementary file 4 [file oncotarget-09-10665-s004.pdf]

Table S3. Candidate protein biomarkers for CRC

Protein IDs	Protein names	Gene names	Log2 FC	NC	CRC	Subcellular localization
P49006	MARCKS-related protein	MARCKSL1	CRC	0	4	Plasma membrane; extracellular exosomes
P22894	Neutrophil collagenase	MMP8	CRC	0	4	Extracellular space
Q9H3R2	Mucin-13	MUC13	CRC	0	4	Extracellular space
O75340	Programmed cell death protein 6	PDCD6	CRC	0	4	Nucleus; endoplasmic reticulum; extracellular exosomes
Q9BVK6	Transmembrane emp24 domain-containing protein 9	TMED9	CRC	0	4	Golgi apparatus; membrane; extracellular exosomes
Q13509	Tubulin beta-3 chain	TUBB3	CRC	0	4	Nucleus; cytosol; membrane; extracellular exosomes
P49913	Cathelicidin antimicrobial peptide	CAMP	CRC	0	4	Extracellular space
Q9Y2V2	Calcium-regulated heat stable protein 1	CARHSP1	CRC	0	4	Cytoplasm; extracellular exosomes
P32320	Cytidine deaminase	CDA	CRC	0	4	Extracellular space
P40199	Carcinoembryonic antigen-related cell adhesion molecule 6	CEACAM6	CRC	0	4	Extracellular space
Q9UHY7	Enolase-phosphatase E1	ENOPH1	CRC	0	4	Nucleus; exracellular exosomes
P28676	Grancalcin	GCA	CRC	0	4	Plasma membrane; extracellular exosomes
P28799	Granulins	GRN	CRC	0	4	Extracellular space
Q53GQ0	Estradiol 17-beta-dehydrogenase 12	HSD17B12	CRC	0	4	Extracellular matrix
P17213	Bactericidal permeability-increasing protein	BPI	1.90	3	4	Extracellular space
P05451	Lithostathine-1-alpha	REG1A	1.74	3	4	Extracellular exosomes
P49588	Alanine--tRNA ligase, cytoplasmic	AARS	1.57	5	4	Mitochondria; extracellular exosomes
Q9NYU2;Q9NYU1	UDP-glucose:glycoprotein glucosyltransferase 1	UGGT1	1.46	3	4	Endoplasmic reticulum; extracellular exosomes
O00487	26S proteasome non-ATPase regulatory subunit 14	PSMD14	1.46	3	4	Proteasome; cytosol; extracellular exosomes
P49368	T-complex protein 1 subunit gamma	CCT3	1.15	6	4	Plasma membrane; extracellular exosomes
P62857	40S ribosomal protein S28	RPS28	1.07	5	4	Cytoplasm; extracellular exosomes
Q99460	26S proteasome non-ATPase regulatory subunit 1	PSMD1	1.06	4	4	Nucleus; cytosol; membrane; extracellular exosomes
P62318	Small nuclear ribonucleoprotein Sm D3	SNRPD3	1.04	3	4	Nucleoplasm; extracellular exosomes
O95793	Double-stranded RNA-binding protein Staufen homolog 1	STAU1	CRC	0	5	Membrane; extracellular exosomes
Q9HB07	UPF0160 protein MYG1, mitochondrial	C12orf10	CRC	0	5	Extracellular exosomes
O14979	Heterogeneous nuclear ribonucleoprotein D-like	HNRNPDL	CRC	0	5	Cytoplasm; extracellular exosomes
P15090;P02689	Fatty acid-binding protein, adipocyte	FABP4	38.73	3	5	Cytoplasm; extracellular exosomes
P49327	Fatty acid synthase	FASN	2.64	5	5	Plasma membrane; Golgi apparatus; extracellular exosomes
P32969	60S ribosomal protein L9	RPL9	2.54	3	5	Cytoplasm; nucleus; extracellular matrix
P24821	Tenascin	TNC	2.53	3	5	Extracellular space
P41250	Glycine--tRNA ligase	GARS	2.37	6	5	Cytoplasm; extracellular exosomes
Q14247	Src substrate cortactin	CTTN	2.02	4	5	Golgi apparatus; extracellular exosomes
Q99829	Copine-1	CPNE1	1.99	4	5	Membrane; extracellular exosomes
Q01813	ATP-dependent 6-phosphofructokinase, platelet type	PFKP	1.91	3	5	Nucleus; membrane; extracellular matrix
P62195;Q8NB90	26S protease regulatory subunit 8	PSMC5	1.86	3	5	Proteasome; nucleus; cytoplasm; extracellular exosomes
P60866	40S ribosomal protein S20	RPS20	1.85	4	5	Membrane; extracellular exosomes
P50454	Serpin H1	SERPINH1	1.84	4	5	Extracellular space
P15880	40S ribosomal protein S2	RPS2	1.73	4	5	Nucleus; membrane; extracellular exosomes
P39023;Q92901	60S ribosomal protein L3	RPL3	1.69	4	5	Nucleus; extracellular exosomes
P14868	Aspartate--tRNA ligase, cytoplasmic	DARS	1.48	6	5	Membrane; extracellular exosomes
P05198	Eukaryotic translation initiation factor 2 subunit 1	EIF2S1	1.47	4	5	Nucleus; exracellular exosomes
P52272	Heterogeneous nuclear ribonucleoprotein M	HNRNPM	1.43	6	5	Membrane; extracellular matrix
P62244	40S ribosomal protein S15a	RPS15A	1.40	4	5	Membrane; extracellular matrix
P41091;Q2VIR3	Eukaryotic translation initiation factor 2 subunit 3	EIF2S3;EIF2S3L	1.31	5	5	Nucleus; exracellular exosomes
P62424	60S ribosomal protein L7a	RPL7A	1.27	3	5	Nucleus; membrane; extracellular exosomes
P50991	T-complex protein 1 subunit delta	CCT4	1.26	6	5	Cytoplasm; extracellular exosomes
O43242	26S proteasome non-ATPase regulatory subunit 3	PSMD3	1.24	4	5	Membrane; extracellular matrix
P21281;P15313	V-type proton ATPase subunit B, brain isoform	ATP6V1B2	1.24	3	5	Plasma membrane; extracellular exosomes
P35268	60S ribosomal protein L22	RPL22	1.23	5	5	Extracellular matrix
Q13630	GDP-L-fucose synthase	TSTA3	1.18	6	5	Cytoplasm; extracellular exosomes
P39019	40S ribosomal protein S19	RPS19	1.06	4	5	Extracellular space
Q16543	Hsp90 co-chaperone Cdc37	CDC37	1.05	4	5	Cytoplasm; extracellular exosomes
O60888	Protein CutA	CUTA	1.04	5	5	Membrane; extracellular exosomes
P62249	40S ribosomal protein S16	RPS16	1.00	4	5	Extracellular matrix
Q01130;Q9BRL6	Serine/arginine-rich splicing factor 2	SRSF2;SRSF8	CRC	0	6	Nucleus; extracellular exosomes
O43491	Band 4.1-like protein 2	EPB41L2	4.65	3	6	Membrane; extracellular exosomes
P14780	Matrix metalloproteinase-9	MMP9	4.64	4	6	Extracellular space
P25815	Protein S100-P	S100P	4.34	3	6	Nucleus; extracellular exosomes
P22234	Phosphoribosylaminoimidazole carboxylase	PAICS	3.29	4	6	Cytoplasm; membrane; extracellular exosomes
P53396	ATP-citrate synthase	ACLY	3.20	6	6	Membrane; extracellular exosomes
Q02790	Peptidyl-prolyl cis-trans isomerase FKBP4	FKBP4	3.08	6	6	Nucleoplasm; extracellular exosomes
P41218	Myeloid cell nuclear differentiation antigen	MNDA	2.65	4	6	Nucleus; extracellular exosomes
O75608	Acyl-protein thioesterase 1	LYPLA1	2.24	5	6	Mitochondria; extracellular exosomes
P31949	Protein S100-A11	S100A11	2.06	5	6	Extracellular space
P23396	40S ribosomal protein S3	RPS3	1.96	6	6	Nucleus; cytosol; extracellular matrix
Q9UKK9	ADP-sugar pyrophosphatase	NUDT5	1.94	5	6	Membrane; extracellular matrix
P55884	Eukaryotic translation initiation factor 3 subunit B	EIF3B	1.90	6	6	Cytoplasm; extracellular exosomes
P61313	60S ribosomal protein L15	RPL15	1.86	5	6	Nucleus; cytosol; extracellular exosomes
Q16531	DNA damage-binding protein 1	DDB1	1.82	6	6	Nucleus; exracellular exosomes
Q15181	Inorganic pyrophosphatase	PPA1	1.74	7	6	Cytoplasm; extracellular exosomes
P00492	Hypoxanthine-guanine phosphoribosyltransferase	HPRT1	1.70	6	6	Cytoplasm; extracellular exosomes
P28062	Proteasome subunit beta type-8	PSMB8	1.70	5	6	Cytoplasm; extracellular exosomes
P53621	Coatomer subunit alpha	COPA	1.69	5	6	Extracellular space
P19338	Nucleolin	NCL	1.69	7	6	Nucleus; membrane; extracellular exosomes
P30740	Leukocyte elastase inhibitor	SERPINB1	1.66	5	6	Extracellular space
P46781	40S ribosomal protein S9	RPS9	1.60	5	6	Nucleus; cytoplasm; membrane; extracellular exosomes
P62701;Q8TD47	40S ribosomal protein S4, X isoform	RPS4X	1.60	7	6	Extracellular matrix; extracellular exosomes
P04179	Superoxide dismutase [Mn], mitochondrial	SOD2	1.59	6	6	Mitochondria; extracellular exosomes
P20618	Proteasome subunit beta type-1	PSMB1	1.57	5	6	Proteasome; extracellular exosomes
P62269	40S ribosomal protein S18	RPS18	1.54	5	6	Nucleus; membrane; extracellular exosomes
O43776	Asparagine--tRNA ligase, cytoplasmic	NARS	1.54	6	6	Cytoplasm; extracellular exosomes
Q9UL46	Proteasome activator complex subunit 2	PSME2	1.47	3	6	Nucleoplasm; cytoplasm; membrane; extracellular exosomes
P36578	60S ribosomal protein L4	RPL4	1.47	6	6	nucleus; extracellular exosomes
P46782	40S ribosomal protein S5;40S ribosomal protein S5, N-terminally processed	RPS5	1.46	5	6	Nucleus; membrane; extracellular matrix
P11216	Glycogen phosphorylase, brain form	PYGB	1.45	7	6	Membrane; extracellular matrix
P48637	Glutathione synthetase	GSS	1.44	5	6	Cytoplasm; extracellular exosomes
P61081	NEDD8-conjugating enzyme Ubc12	UBE2M	1.41	3	6	Cytoplasm; extracellular exosomes
P26599	Polypyrimidine tract-binding protein 1	PTBP1	1.39	6	6	Nucleus; membrane; extracellular exosomes
P62277	40S ribosomal protein S13	RPS13	1.39	7	6	Extracellular matrix
Q15075	Early endosome antigen 1	EEA1	1.35	4	6	Cytoplasm; extracellular exosomes
Q07955	Serine/arginine-rich splicing factor 1	SRSF1	1.34	7	6	Nucleus; extracellular exosomes
Q00839	Heterogeneous nuclear ribonucleoprotein U	HNRNPU	1.30	7	6	Nucleus; extracellular matrix
P13861;P31323	cAMP-dependent protein kinase type II-alpha regulatory subunit	PRKAR2A	1.29	6	6	Plasma membrane; extracellular exosomes

Q14103	Heterogeneous nuclear ribonucleoprotein D0	HNRNPD	1.29	7	6	Nucleus; extracellular exosomes
P13693	Translationally-controlled tumor protein	TPT1	1.26	5	6	Extracellular space
P63244	Guanine nucleotide-binding protein subunit beta-2-like 1	GNB2L1	1.25	7	6	Nucleus; membrane; extracellular matrix
P62081	40S ribosomal protein S7	RPS7	1.24	6	6	Nucleus; membrane; extracellular matrix
Q14204	Cytoplasmic dynein 1 heavy chain 1	DYNC1H1	1.24	6	6	Membrane; extracellular matrix
P06753	Tropomyosin alpha-3 chain	TPM3	1.22	7	6	Cytoplasm; extracellular exosomes
P08865	40S ribosomal protein SA	RPSA	1.19	6	6	Membrane; extracellular exosomes
P62826	GTP-binding nuclear protein Ran	RAN	1.18	7	6	Membrane; extracellular matrix
P62888	60S ribosomal protein L30	RPL30	1.16	7	6	Membrane; extracellular matrix
P05388;Q8NHW5	60S acidic ribosomal protein P0	RPLP0;RPLP0P6	1.15	5	6	Nucleus; extracellular exosomes
P62993	Growth factor receptor-bound protein 2	GRB2	1.14	4	6	Golgi apparatus; membrane; extracellular exosomes
P60900	Proteasome subunit alpha type-6	PSMA6	1.13	7	6	Nucleus; extracellular exosomes
Q9UQ80	Proliferation-associated protein 2G4	PA2G4	1.12	7	6	Nucleus; membrane; extracelluar exosomes
P05387	60S acidic ribosomal protein P2	RPLP2	1.12	7	6	Cytoplasm; membrane; extracellular exosomes
O00299	Chloride intracellular channel protein 1	CLIC1	1.11	7	6	Extracellular space
Q15366;P57721	Poly(rC)-binding protein 2	PCBP2	1.11	4	6	Nucleus; membrane; extracelluar exosomes
P31939	Bifunctional purine biosynthesis protein PURH	ATIC	1.11	7	6	Membrane; extracellular exosomes
P50990	T-complex protein 1 subunit theta	CCT8	1.11	6	6	Mitochondria; extracellular exosomes
P25786	Proteasome subunit alpha type-1	PSMA1	1.11	5	6	Proteasome; cytosol; extracellular exosomes
O60547	GDP-mannose 4,6 dehydratase	GMDS	1.11	6	6	Cytoplasm; extracellular exosomes
P11586	C-1-tetrahydrofolate synthase, cytoplasmic	MTHFD1	1.11	6	6	Mitochondria; extracellular exosomes
P32455	Interferon-induced guanylate-binding protein 1	GBP1	1.08	5	6	Extracellular space
P50570;Q9UQ16	Dynamin-2	DNM2	1.05	3	6	Mitochondria; extracellular exosomes
P46109	Crk-like protein	CRKL	1.01	3	6	Endosome; extracellular exosomes
P13611	Versican core protein	VCAN	39.34	3	7	Extracellular matrix
P08246	Neutrophil elastase	ELANE	3.28	5	7	Extracellular space
P06731	Carcinoembryonic antigen-related cell adhesion molecule 5	CEACAM5	3.18	6	7	Plasma membrane; extracellular exosomes
P20160	Azurocidin	AZU1	3.18	6	7	Extracellular space
P05164	Myeloperoxidase	MPO	2.93	7	7	Extracellular space
P80188	Neutrophil gelatinase-associated lipocalin	LCN2	2.69	6	7	Extracellular space
Q15046	Lysine--tRNA ligase	KARS	2.56	5	7	Extracellular space
P02788	Lactotransferrin	LTF	2.49	7	7	Extracellular space
P35754	Glutaredoxin-1	GLRX	2.32	4	7	Nucleus; cytosol; extracellular exosomes
P53999	Activated RNA polymerase II transcriptional coactivator p15	SUB1	2.19	5	7	Nucleus; extracellular exosomes
Q13162	Peroxiredoxin-4	PRDX4	2.10	5	7	Extracellular space
Q15582	Transforming growth factor-beta-induced protein ig-h3	TGFBI	2.09	6	7	Extracellular space
P06702	Protein S100-A9	S100A9	2.05	7	7	Extracellular space
P56537	Eukaryotic translation initiation factor 6	EIF6	1.92	6	7	Nucleus; exracellular exosomes
P61626	Lysozyme C	LYZ	1.90	7	7	Extracellular space
P14866	Heterogeneous nuclear ribonucleoprotein L	HNRNPL	1.84	7	7	Nucleus; extracellular exosomes
P05109	Protein S100-A8	S100A8	1.81	7	7	Extracellular space
P51858	Hepatoma-derived growth factor	HDGF	1.79	6	7	Extracellular space
P02794	Ferritin heavy chain	FTH1	1.71	7	7	Nucleus; cytosol; extracellular matrix
P26583	High mobility group protein B2	HMGB2	1.70	7	7	Extracellular space
P05783;CON__H-INV:I	Keratin, type I cytoskeletal 18	KRT18	1.69	7	7	Cytoplasm; extracellular exosomes
P61604	10 kDa heat shock protein	HSPE1	1.66	7	7	Mitochondria; extracellular exosomes
P30050	60S ribosomal protein L12	RPL12	1.57	6	7	Nucleolus; cytosol; extracellular matrix
P37802	Transgelin-2	TAGLN2	1.56	7	7	Extracellular exosomes
P09651;Q32P51	Heterogeneous nuclear ribonucleoprotein A1	HNRNPA1;HNRNPA1L2	1.54	7	7	Nucleus; membrane; extracellular exosomes
P59666;P59665	Neutrophil defensin 3	DEFA3;DEFA1	1.49	7	7	Extracellular space
P23246	Splicing factor, proline- and glutamine-rich	SFPQ	1.49	5	7	Nucleus; extracellular matrix
P09429;B2RPK0	High mobility group protein B1	HMGB1	1.43	7	7	Extracellular space
P40121	Macrophage-capping protein	CAPG	1.42	7	7	Nucleus; exracellular exosomes
P26641	Elongation factor 1-gamma	EEF1G	1.41	7	7	Nucleus; exracellular exosomes
P67936	Tropomyosin alpha-4 chain	TPM4	1.39	6	7	Membrane; extracellular exosomes
P09525	Annexin A4	ANXA4	1.37	6	7	Nucleus; membrane; extracellular exosomes
Q9BRA2	Thioredoxin domain-containing protein 17	TXNDC17	1.36	6	7	Cytoplasm; extracellular exosomes
P23284	Peptidyl-prolyl cis-trans isomerase B	PPIB	1.34	7	7	Nucleus; endoplasmic reticulum; extracellular exosomes
P14174	Macrophage migration inhibitory factor	MIF	1.34	7	7	Extracellular space
O75223	Gamma-glutamylcyclotransferase	GGCT	1.32	4	7	Cytoplasm; extracellular exosomes
P12724	Eosinophil cationic protein	RNASE3	1.31	7	7	Extracellular space
P02792	Ferritin light chain	FTL	1.30	6	7	Nucleus; membrane; extracellular exosomes
P07900;Q14568;Q58F	Heat shock protein HSP 90-alpha	HSP90AA1	1.30	7	7	Extracellular space
P52566	Rho GDP-dissociation inhibitor 2	ARHGDIB	1.30	7	7	Membrane; extracellular exosomes
P07910	Heterogeneous nuclear ribonucleoproteins C1/C2	HNRNPC	1.29	6	7	Nucleus; membrane; extracellular exosomes
Q01469;A8MUU1	Fatty acid-binding protein, epidermal	FABP5	1.24	7	7	Cytoplasm; extracellular exosomes
P19105;O14950	Myosin regulatory light chain 12A;Myosin regulatory light chain 12B	MYL12A;MYL12B	1.23	5	7	Cytoplasm; extracellular exosomes
P08311	Cathepsin G	CTSG	1.20	7	7	Extracellular space
Q15185	Prostaglandin E synthase 3	PTGES3	1.18	7	7	Nucleus; extracellular exosomes
P62805	Histone H4	HIST1H4A	1.16	7	7	Extracellular space
P14625;Q58FF3	Endoplasmin	HSP90B1	1.13	7	7	Extracellular space
P22626	Heterogeneous nuclear ribonucleoproteins A2/B1	HNRNPA2B1	1.13	7	7	Membrane; extracellular exosomes
P61978	Heterogeneous nuclear ribonucleoprotein K	HNRNPK	1.12	7	7	Extracellular matrix
P37837	Transaldolase	TALDO1	1.09	7	7	Nucleus; extracellular exosomes
P13639	Elongation factor 2	EEF2	1.08	7	7	Extracellular matrix
P62258	14-3-3 protein epsilon	YWHAE	1.05	7	7	Mitochondria; extracellular exosomes
Q15084	Protein disulfide-isomerase A6	PDIA6	1.02	5	7	Endoplasmic reticulum; membrane; extracellular exosomes
P00924	Enolase 1	ENO1	1.02	7	7	Extracellular space

FC: fold change; NC: Normal Colon; CRC:Colorectal cancer
