# Supplementary material for: Innovative methodology for the identification of soluble biomarkers in fresh tissues
Source: Oncotarget. 2018 Jan 31;9(12):10665–80. doi: 10.18632/oncotarget.24366 (PMC5828218; doi:10.18632/oncotarget.24366)
Supplement: Supplementary file 5 [file oncotarget-09-10665-s005.pdf]

Table S4. Candidate protein biomarkers for CRC-LM

Protein IDs	Protein names	Gene names	Log2 FC	NL	CRC-LM	Subcellular localization
P02749	Beta-2-glycoprotein 1	APOH	1.00	6	6	Extracellular Space
P67936	Tropomyosin alpha-4 chain	TPM4	1.01	6	6	Cytoplasm; extracellular exosomes
P08246	Neutrophil elastase	ELANE	1.01	6	6	Extracellular Space
P35237	Serpin B6	SERPINB6	1.01	4	6	Cytoplasm; extracellular exosomes
P02787	Serotransferrin	TF	1.02	6	6	Extracellular Space
P02647	Apolipoprotein A-I	APOA1	1.02	6	6	Extracellular Space
P07225	Vitamin K-dependent protein S	PROS1	1.04	4	6	Extracellular Space
P04196	Histidine-rich glycoprotein	HRG	1.06	6	6	Extracellular Space
Q96QK1	Vacuolar protein sorting-associated protein 35	VPS35	1.07	6	6	Cytoplasm; extracellular exosomes
P00751	Complement factor B	CFB	1.11	6	6	Extracellular Space
P02763	Alpha-1-acid glycoprotein 1	ORM1	1.11	6	6	Extracellular Space
P68366;A6NHL2	Tubulin alpha-4A chain	TUBA4A	1.12	6	6	Extracellular Space
P59666;P59665	Neutrophil defensin 3;HP 3-56	DEFA3;DEFA1	1.13	6	6	Extracellular Space
P80188	Neutrophil gelatinase-associated lipocalin	LCN2	1.13	6	6	Extracellular Space
P63092;Q5JWF2;P38405	Guanine nucleotide-binding protein G(s) subunit alpha isoforms short	GNAS	1.14	5	5	Plasma Membrane; extracellular exosomes
P04179	Superoxide dismutase [Mn], mitochondrial	SOD2	1.14	4	6	Cytoplasm; extracellular exosomes
P02675	Fibrinogen beta chain	FGB	1.14	6	6	Extracellular Space
Q9Y3B3	Transmembrane emp24 domain-containing protein 7	TMED7	1.14	3	4	Cytoplasm; extracellular exosomes
P00748	Coagulation factor XII	F12	1.14	5	6	Extracellular Space
P46926;Q8TDQ7	Glucosamine-6-phosphate isomerase 1	GNPDA1	1.15	4	5	Cytoplasm; extracellular exosomes
P50914	60S ribosomal protein L14	RPL14	1.16	6	6	Membrane; extracellular exosomes
P01008	Antithrombin-III	SERPINC1	1.16	6	6	Extracellular Space
P02765	Alpha-2-HS-glycoprotein	AHSG	1.16	6	6	Extracellular Space
P15880	40S ribosomal protein S2	RPS2	1.18	6	6	Membrane; extracellular exosomes
P13284	Gamma-interferon-inducible lysosomal thiol reductase	IFI30	1.21	5	6	Extracellular Space
P61626	Lysozyme C	LYZ	1.22	6	6	Extracellular Space
P04843	Dolichyl-diphosphooligosaccharide--protein glycosyltransferase subunit 1	RPN1	1.24	6	6	Extracellular Space
P25311	Zinc-alpha-2-glycoprotein	AZGP1	1.24	6	6	Extracellular Space
P07585	Decorin	DCN	1.24	6	5	Extracellular Space
P02760	Protein AMBP	AMBP	1.25	6	6	Extracellular Space
P06702	Protein S100-A9	S100A9	1.30	5	6	Extracellular Space
Q9Y263	Phospholipase A-2-activating protein	PLAA	1.31	4	5	Extracellular space
P63104	14-3-3 protein zeta/delta	YWHAZ	1.32	6	6	Cytoplasm; extracellular exosomes
Q9NYU2	UDP-glucose:glycoprotein glucosyltransferase 1	UGGT1	1.34	6	6	Cytoplasm; extracellular exosomes
P02746	Complement C1q subcomponent subunit B	C1QB	1.35	5	6	Extracellular Space
P10909	Clusterin	CLU	1.35	6	6	Extracellular space
Q9UBQ0	Vacuolar protein sorting-associated protein 29	VPS29	1.36	4	6	Cytoplasm; extracellular exosomes
P02679	Fibrinogen gamma chain	FGG	1.36	6	6	Extracellular Space
P29622	Kallistatin	SERPINA4	1.36	5	6	Extracellular Space
O14908	PDZ domain-containing protein GIPC1	GIPC1	1.40	4	6	Cytoplasm; extracellular exosomes
P41218	Myeloid cell nuclear differentiation antigen	MNDA	1.42	5	6	Nucleus; exracellular exosomes
P21333	Filamin-A	FLNA	1.44	6	6	Extracellular Space
P02790	Hemopexin	HPX	1.46	6	6	Extracellular Space
O43852	Calumenin	CALU	1.48	5	6	Extracellular Space
P05452	Tetranectin	CLEC3B	1.50	5	6	Extracellular Space
P41252	Isoleucine--tRNA ligase, cytoplasmic	IARS	1.50	6	6	Cytoplasm; extracellular exosomes
P02766	Transthyretin	TTR	1.52	6	6	Extracellular Space
P51884	Lumican	LUM	1.52	6	6	Extracellular Space
P01011	Alpha-1-antichymotrypsin;Alpha-1-antichymotrypsin His-Pro-less	SERPINA3	1.55	6	6	Extracellular Space
P11216	Glycogen phosphorylase, brain form	PYGB	1.57	6	6	Cytoplasm; membrane ; extracellular exosomes
P19652	Alpha-1-acid glycoprotein 2	ORM2	1.60	6	6	Extracellular Space
Q9NZ08	Endoplasmic reticulum aminopeptidase 1	ERAP1	1.62	4	6	Extracellular Space
P09486	SPARC	SPARC	1.63	3	5	Extracellular Space
P01019	Angiotensinogen	AGT	1.64	6	6	Extracellular Space
P07357	Complement component C8 alpha chain	C8A	1.67	6	6	Extracellular Space
Q14520	Hyaluronan-binding protein 2	HABP2	1.67	4	6	Extracellular Space
Q86UX7	Fermitin family homolog 3	FERMT3	1.71	6	6	Cytoplasm; extracellular exosomes
P01009	Alpha-1-antitrypsin	SERPINA1	1.72	6	6	Extracellular Space
P02774	Vitamin D-binding protein	GC	1.75	6	6	Extracellular Space
Q9Y6R7	IgGFc-binding protein	FCGBP	1.77	5	5	Extracellular Space
P49411	Elongation factor Tu, mitochondrial	TUFM	1.79	6	6	extracellular exosomes
P29966	Myristoylated alanine-rich C-kinase substrate	MARCKS	1.83	5	6	Plasma Membrane; extracellular exosomes
P07360	Complement component C8 gamma chain	C8G	1.85	6	6	Extracellular Space
P35613	Basigin	BSG	1.86	5	5	Extracellular space
Q12805	EGF-containing fibulin-like extracellular matrix protein 1	EFEMP1	1.86	6	6	Extracellular Space
P07195	L-lactate dehydrogenase B chain	LDHB	1.87	6	6	Cytoplasm; extracellular exosomes
P07358	Complement component C8 beta chain	C8B	1.89	5	6	Extracellular Space
P98160	Basement membrane-specific heparan sulfate proteoglycan core protein	HSPG2	1.91	6	6	Extracellular Space
P00747	Plasminogen	PLG	1.91	6	6	Extracellular Space
Q7Z6Z7	E3 ubiquitin-protein ligase HUWE1	HUWE1	1.93	6	6	Nucleus; extracellulare exosomes
P06727	Apolipoprotein A-IV	APOA4	1.93	6	6	Extracellular Space
P07602	Proactivator polypeptide	PSAP	1.95	6	6	Extracellular Space
P10643	Complement component C7	C7	1.99	6	6	Extracellular Space
Q01813	6-phosphofructokinase type C	PFKP	2.00	6	6	Cytoplasm; extracellular matrix
P12109	Collagen alpha-1(VI) chain	COL6A1	2.02	4	6	Extracellular Space
P11177	Pyruvate dehydrogenase E1 component subunit beta, mitochondrial	PDHB	2.05	6	6	Nucleus; extracellular exosomes
Q00610	Clathrin heavy chain 1	CLTC	2.08	6	6	Plasma Membrane; extracellular matrix
P15151	Poliovirus receptor	PVR	2.09	4	6	Extracellular Space
P06396	Gelsolin	GSN	2.14	6	6	Extracellular Space
P30740	Leukocyte elastase inhibitor	SERPINB1	2.15	6	6	Cytoplasm; extracellular exosomes
P21810	Biglycan	BGN	2.17	6	6	Extracellular Space
P08670;P17661;Q16352;P07197	Vimentin	VIM	2.23	6	6	Cytoplasm; extracellular space
P24821	Tenascin	TNC	2.26	5	6	Extracellular Space
P00488	Coagulation factor XIII A chain	F13A1	2.31	3	5	Extracellular Space
Q9Y265	RuvB-like 1	RUVBL1	2.32	6	6	Nucleus; extracellular exosomes
P60903	Protein S100-A10	S100A10	2.38	6	6	Extracellular Space
Q15582	Transforming growth factor-beta-induced protein ig-h3	TGFBI	2.44	6	6	Extracellular Space
Q9NZU5	LIM and cysteine-rich domains protein 1	LMCD1	2.50	3	5	Extracellular Space
Q9BVK6	Transmembrane emp24 domain-containing protein 9	TMED9	2.56	4	5	Cytoplasm; extracellular exosomes
P18428	Lipopolysaccharide-binding protein	LBP	2.66	4	6	Extracellular Space
P06703	Protein S100-A6	S100A6	2.67	6	6	Cytoplasm; extracellular exosomes
P50454	Serpin H1	SERPINH1	2.67	5	6	Extracellular Space
Q9P2R7	Succinyl-CoA ligase [ADP-forming] subunit beta, mitochondrial	SUCLA2	2.72	5	5	Cytoplasm; extracellular exosomes
Q01469;A8MUU1	Fatty acid-binding protein, epidermal	FABP5	2.79	6	6	Cytoplasm; extracellular exosomes

P02751	Fibronectin	FN1	2.83	6	6	Extracellular Space
O00299	Chloride intracellular channel protein 1	CLIC1	2.87	6	6	Extracellular Space
Q15113	Procollagen C-endopeptidase enhancer 1	PCOLCE	2.89	4	6	Extracellular Space
Q92598	Heat shock protein 105 kDa	HSPH1	2.90	6	6	Extracellular Space
P22695	Cytochrome b-c1 complex subunit 2, mitochondrial	UQCRC2	2.93	6	6	Mitochondria; extracellular exosomes
P12004	Proliferating cell nuclear antigen	PCNA	2.95	6	6	Nucleus; extracellular exosomes
P14618	Pyruvate kinase isozymes M1/M2	PKM	3.01	6	6	Cytoplasm; extracellular matrix
Q14195	Dihydropyrimidinase-related protein 3	DPYSL3	3.10	6	6	Extracellular Space
P12111	Collagen alpha-3(VI) chain	COL6A3	3.26	4	6	Extracellular Space
Q7Z406	Myosin-14	MYH14	3.30	6	6	Extracellular Space
P02748;CON__Q3MHN2	Complement component C9	C9	3.36	6	6	Extracellular Space
P17844	Probable ATP-dependent RNA helicase DDX5	DDX5	3.37	4	6	Extracellular matrix
P12277	Creatine kinase B-type	CKB	3.63	6	6	Extracellular Space
Q12931	Heat shock protein 75 kDa, mitochondrial	TRAP1	3.75	4	6	Cytoplasm; extracellular exosomes
P07996	Thrombospondin-1	THBS1	3.82	6	6	Extracellular Space
Q15149	Plectin	PLEC	3.88	6	6	Cytoplasm; extracellular exosomes
P09327;O75366	Villin-1	VIL1	4.03	5	6	Cytoplasm; extracellular exosomes
P17931	Galectin-3	LGALS3	4.06	5	6	Extracellular Space
Q99715	Collagen alpha-1(XII) chain	COL12A1	4.10	5	5	Extracellular Space
Q9HC84;P98088	Mucin-5B	MUC5B	4.29	4	5	Extracellular Space
Q14764	Major vault protein	MVP	4.72	6	6	Nucleus; extracellular exosomes
P78527	DNA-dependent protein kinase catalytic subunit	PRKDC	5.20	6	6	Extracellular matrix; nucleus
Q8IUX7	Adipocyte enhancer-binding protein 1	AEBP1	5.80	5	6	Extracellular Space
P01033	Metalloproteinase inhibitor 1	TIMP1	6.87	4	6	Extracellular Space
P23921	Ribonucleoside-diphosphate reductase large subunit	RRM1	CRC-LM	0	3	Nucleus;extracellular exosomes
Q92520	Protein FAM3C	FAM3C	CRC-LM	0	3	Extracellular Space
P15169	Carboxypeptidase N catalytic chain	CPN1	CRC-LM	0	3	Extracellular Space
P36222	Chitinase-3-like protein 1	CHI3L1	CRC-LM	0	3	Extracellular Space
Q15813	Tubulin-specific chaperone E	TBCE	CRC-LM	0	3	Cytoplasm; extracellular exosomes
O94919	Endonuclease domain-containing 1 protein	ENDOD1	CRC-LM	0	3	Extracellular Space
O14828	Secretory carrier-associated membrane protein 3	SCAMP3	CRC-LM	0	3	Cytoplasm; extracellular exosomes
Q14254	Flotillin-2	FLOT2	CRC-LM	0	3	Plasma Membrane; extracellular exosomes
Q14344	Guanine nucleotide-binding protein subunit alpha-13	GNA13	CRC-LM	0	3	Plasma Membrane; extracellular exosomes
O43657	Tetraspanin-6	TSPAN6	CRC-LM	0	3	Plasma Membrane; extracellular exosomes
Q9H9S4	Calcium-binding protein 39-like	CAB39L	CRC-LM	0	3	Cytoplasm; extracellular exosomes
A3KMH1	von Willebrand factor A domain-containing protein 8	VWA8	CRC-LM	0	3	Extracellular Space
Q9Y5X1	Sorting nexin-9	SNX9	CRC-LM	0	3	Cytoplasm; extracellular exosomes
P62070	Ras-related protein R-Ras2	RRAS2	19.51	3	5	Plasma Membrane; extracellular exosomes
O43278	Kunitz-type protease inhibitor 1	SPINT1	CRC-LM	0	3	Extracellular Space
P33241	Lymphocyte-specific protein 1	LSP1	CRC-LM	0	3	Plasma Membrane; extracellular exosomes
Q9BWS9	Chitinase domain-containing protein 1	CHID1	CRC-LM	0	3	Extracellular Space
Q06828	Fibromodulin	FMOD	CRC-LM	0	3	Extracellular Space
P41222	Prostaglandin-H2 D-isomerase	PTGDS	CRC-LM	0	3	Extracellular Space
Q9NRN5	Olfactomedin-like protein 3	OLFML3	CRC-LM	0	3	Extracellular Space
P13473	Lysosome-associated membrane glycoprotein 2	LAMP2	CRC-LM	0	3	Extracellular Space
Q9UGM5	Fetuin-B	FETUB	CRC-LM	0	3	Extracellular Space
Q9NR99	Matrix-remodeling-associated protein 5	MXRA5	CRC-LM	0	3	Extracellular Space
Q9NQC3	Reticulon-4	RTN4	20.24	3	6	Plasma Membrane; extracellular exosomes
O43760	Synaptogyrin-2	SYNGR2	CRC-LM	0	3	Cytoplasm; extracellular exosomes
Q8TD06	Anterior gradient protein 3 homolog	AGR3	CRC-LM	0	3	Extracellular Space
O96008	Mitochondrial import receptor subunit TOM40 homolog	TOMM40	CRC-LM	0	3	Cytoplasm; extracellular exosomes
Q96BQ1	Protein FAM3D	FAM3D	CRC-LM	0	3	Extracellular Space
Q16610	Extracellular matrix protein 1	ECM1	20.75	3	6	Extracellular Space
Q15437	Protein transport protein Sec23B	SEC23B	20.76	3	6	Extracellular Space
Q07654	Trefoil factor 3	TFF3	CRC-LM	0	3	Extracellular Space
P17900	Ganglioside GM2 activator	GM2A	20.95	3	6	Mitochondria; extracellular exosomes
Q16819	Meprin A subunit alpha	MEP1A	22.22	3	6	Extracellular Space
Q96TA1	Niban-like protein 1	FAM129B	22.35	3	6	Cytoplasm; extracellular exosomes
P00746	Complement factor D	CFD	23.06	3	6	Extracellular Space
P45880	Voltage-dependent anion-selective channel protein 2	VDAC2	23.32	3	5	Cytoplasm; extracellular exosomes
P31949	Protein S100-A11	S100A11	23.59	3	6	Extracellular Space
O95994	Anterior gradient protein 2 homolog	AGR2	23.71	3	6	Extracellular Space
P24593	Insulin-like growth factor-binding protein 5	IGFBP5	CRC-LM	0	4	Extracellular Space
Q9ULE6	Paladin	PALD1	CRC-LM	0	4	Cytoplasm; extracellular exosomes
P14543	Nidogen-1	NID1	CRC-LM	0	4	Extracellular Space
P04155	Trefoil factor 1	TFF1	CRC-LM	0	4	Extracellular Space
P48960	CD97 antigen	CD97	CRC-LM	0	6	Extracellular Space
Q9BVP2	Guanine nucleotide-binding protein-like 3	GNL3	CRC-LM	0	5	Extracellular Space
Q9H2P0	Activity-dependent neuroprotector homeobox protein	ADNP	CRC-LM	0	5	Extracellular Space
Q6UX71	Plexin domain-containing protein 2	PLXDC2	CRC-LM	0	5	Extracellular Space
P07942	Laminin subunit beta-1	LAMB1	CRC-LM	0	6	Extracellular Space
P51911	Calponin-1	CNN1	CRC-LM	0	4	Cytoplasm; membrane; extracellular exosomes
Q10713	Mitochondrial-processing peptidase subunit alpha	PMPCA	CRC-LM	0	4	Extracellular space
P20908	Collagen alpha-1(V) chain	COL5A1	CRC-LM	0	4	Extracellular Space
Q92599	Septin-8	SEPT8	CRC-LM	0	5	Extracellular Space
P02461	Collagen alpha-1(III) chain	COL3A1	CRC-LM	0	5	Extracellular Space
P61803	Dolichyl-diphosphooligosaccharide--protein glycosyltransferase subunit DAD1	DAD1	CRC-LM	0	5	Cytoplasm; membrane; extracellular exosomes
P11047	Laminin subunit gamma-1	LAMC1	CRC-LM	0	5	Extracellular Space
Q14767	Latent-transforming growth factor beta-binding protein 2	LTBP2	CRC-LM	0	4	Extracellular Space
Q96CG8	Collagen triple helix repeat-containing protein 1	CTHRC1	CRC-LM	0	4	Extracellular Space
P34096	Ribonuclease 4	RNASE4	CRC-LM	0	5	Extracellular Space
P16444	Dipeptidase 1	DPEP1	CRC-LM	0	5	Extracellular Space
Q8TE67	Epidermal growth factor receptor kinase substrate 8-like protein 3	EPS8L3	CRC-LM	0	5	Extracellular Space
P49747	Cartilage oligomeric matrix protein	COMP	CRC-LM	0	6	Extracellular Space
Q8NFJ5	Retinoic acid-induced protein 3	GPRC5A	CRC-LM	0	5	Plasma Membrane; extracellular exosomes
O00391	Sulfhydryl oxidase 1	QSOX1	CRC-LM	0	6	Extracellular space
P55058	Phospholipid transfer protein	PLTP	CRC-LM	0	4	Extracellular Space
P25815	Protein S100-P	S100P	CRC-LM	0	5	Cytoplasm; extracellular exosomes

FC: fold change; NL: Normal Liver; CRC-LM: Colorectal cancer Liver Metastasis
